# Low-Dose Tacrolimus Prevents Dysregulated Peri-Conceptional Ovarian and Systemic Immune Cellular Homeostasis in Subjects with PCOS

**DOI:** 10.1038/s41598-019-42960-x

**Published:** 2019-04-25

**Authors:** Ahmad J. H. Albaghdadi, Carolyn Ann Feeley, Frederick W. K. Kan

**Affiliations:** Department of Biomedical and Molecular Sciences, Faculty of Health Sciences, Queen’s University, Kingston, Ontario, K7L 3N6 Canada

**Keywords:** Experimental models of disease, Preclinical research

## Abstract

Polycystic ovary syndrome (PCOS) is characterized by failure of ovulation and is associated with obesity and chronic inflammation. Recent evidence suggests that anomalous activation of ovarian macrophages and numerical and functional deficits in the Th17 (CD4+IL17A+) and the CD4+CD25+CD127^low^ Tregs plays crucial role in PCOS. We have shown that the pre-pregnancy use of tacrolimus prevents adverse reproductive outcomes in a mouse model of PCOS. Here we used the HFD-NONcNZO mice to test a hypothesized beneficial use of tacrolimus relative to metformin in favorably influencing the ovarian and systemic immune milieux conducive to gestational success in subjects with PCOS. Compared to normative controls, our data revealed an aberrant peri-conceptional suppression of the CD4^+^CD25^+^CD127^low^ Tregs together with an overexpression of the Th17 T cells and lack of coordinated activation of ovarian macrophages in untreated HFD-dNONcNZO mice. Significant variances in treatment outcomes favoured the use of tacrolimus over metformin in treated mice. Consistent with the human fertility studies, this investigation reveals a beneficial systemic use of tacrolimus (0.1 mg/kg) in promoting early pregnancy in individuals with PCOS and suggests the need for further research into the selective inhibition of IL17A as a plausibly alternative immunotherapeutic approach in the clinical management of infertile individuals with PCOS.

## Introduction

Polycystic ovarian syndrome (PCOS) is one of the main causes of anovulatory infertility affecting 5–15% of women of reproductive age^1^. In its typical form, this multi-systemic hormonal disorder is characterized by oligo and/or anovulation, and is often associated with obesity, insulin resistance and chronic inflammation and may constitute a risk factor for ovarian cancer development^2–5^. Physiologically, ovulation is a controlled, temporally restricted inflammatory response mediated by the effector arm of the follicular immune system. Studies showed that cross-talks between resident white blood cells, such as macrophages with systemic T helper cells [Th1 (CD4^+^ IFNγ^+^) and Th2 (CD4^+^IL4^+^)] are critically involved in the process of expulsing the oocyte from the antral/Graafian follicles^6^. Recently, certain immunological deficits have been recognized in women and in animal models favoring an autoimmune etiology in the pathogenesis of PCOS^7–9^. Defective expansion of the CD4^+^CD25^+^CD127^low^ regulatory T-cells (Tregs) at the follicular phase subsequent to inherent aberrancies in Interleukin 2 (IL2) signaling together with dysfunctional production of IL1α and ILβ and elevated serum biomarkers of oxidative stress and auto-antibodies have been reported in women with PCOS^7,10–12^. Altered activation of ovarian macrophages with aberrantly high M1:M2 macrophage ratios in the antral and pre-ovulatory follicles has been reported in a rat model of the 5α-dihydrotestosterone (DHT)-induced PCOS^13^. The inflammatory actions of M1 macrophages can lead to serious tissue damage^14–16^ whereas M2 macrophages are involved in Th2 responses promoting tissue repair and remodeling, cell proliferation, and angiogenesis^17,18^. Additionally, an emerging body of evidence implicates a putative pathogenic role for IL17A in mediating autoimmune and reproductive disturbances reported in women with PCOS (reviewed in^19^). IL-17A is the signature cytokine of the newly identified T helper 17 (Th17) subsets and a pleotropic cytokine member of the IL17 family which includes IL17E and IL17F^20^. Studies showed that these proteins are critical players in host defense and inflammatory disease regulating interactions between adaptive and innate immunity^20–23^. IL17A acts fundamentally by inducing the gene expression for the pro-inflammatory mediators IL1, IL2, TNFα, IFNγ and GM-CSF as well as matrix metalloproteinases and transcriptional factor nuclear κB (NFκB) provoking a potent inflammatory response^24^.

Current therapies for PCOS include oral contraceptives, anti-androgens, drugs which induce ovulation, and metformin^25^. Metformin is a broadly prescribed drug used to reduce insulin levels and functions by interfering with hepatic glucose release and inhibiting androgen production in the ovaries^25,26^. Importantly, emerging evidence revealed significant immunosuppressive properties of metformin when used at high dosage in the treatment of immune-mediated reproductive disorders and in cancer immunotherapy when used in combination with macrolide immunosuppressants such as tacrolimus and/or sirolimus^27,28^. Tacrolimus (FK506) is a 822 kDa lipophilic macrolide lactone antibiotic with potent immune suppressive activity which has recently been used to treat female infertility in obese and diabetic mice^29^ and women with recurrent implantation failure with elevated systemic Th1 (CD4^+^ IFNγ^+^): Th2 (CD4^+^IL4^+^) cell ratios^30^. Tacrolimus inhibits Ca^++^ dependent activation of NFκB and the nuclear factor of activated T cells (NFAT) in T lymphocytes blocking T-cell receptor-mediated lymphokine gene transcription, degranulation, exocytosis and apoptosis^31,32^. Prominently, tacrolimus suppresses T-cell release of INFγ and IL2 as well as TNFα and GM-CSF in activated human plasmacytoid dendritic cells (PCDs) and peripheral blood monocytes (PBMCs) in a dose-dependent manner *in vitro*^33,34^. However, at a low dosage of 0.1 mg/kg, tacrolimus favors the expansion of CD4^+^CD25^+^ Tregs in *ex vivo* cultured human PBMCs^35^.

We have previously reported on the beneficial use of tacrolimus in mitigating severity and incidence of diabesity-associated maternal and fetal gestational adversities in the high-fat fed New Zealand Obese (HFD-dNONcNZO) mice^28^. This mouse is a polygenic model of obesity-induced poor breeding performance with insulin resistance and hyperestrogenemia^36^. Among key contributing factors to subfertility in this mouse lineage are altered ovarian structure and function that are suggestive of PCOS^8^. Therefore, in an attempt to elucidate the mechanism of action of tacrolimus in supporting early gestation in obese and the diabetic subjects with PCOS, the present study was designed to assess the effects of immunosuppression with tacrolimus in comparison with metformin on the activation profile of ovarian macrophages and associated ovarian morphology. Furthermore, given the pathogenic contributions of Th1 and Th17 cells in PCOS^37^, the present study also analyzed the effects of the systemic use of tacrolimus compared to metformin on the peri-conceptional ratios and frequencies of circulating Th1 (CD4^+^IFNγ^+^), Th2 (CD4^+^IL4^+^), Th17 (CD4^+^IL17A^+^) and the CD4^+^CD25^+^CD127^low^ regulatory T cells in the HFD-dNONcNZO mice. Results obtained from the present study support the systemic use of low-dosage tacrolimus (0.1 mg/kg) in the prevention of dysregulated peri-conceptional systemic and ovarian immune cellular homeostasis during early gestation in subjects with PCOS.

## Materials and Methods

### Mouse models

A total of ninety female New Zealand Obese NONcNZO10/LtJ (NZO) mice (004456, Jackson Laboratory, ME, USA) were weaned and maintained on a 60% kCal high-fat diet (HFD) (D12492, Research Diets Inc., NJ, USA) until the age of 21 weeks (Supplemental Fig. S1) and were used as a mouse model of obesity-induced T2DM and PCOS. Twenty female NONcNZO10/LtJ mice were weaned and fed on 6% fat diet high in protein (20% fortified protein pellet diet) (D12450B, Research Diets, New Brunswick, NJ) and were used as normative control cohort (also referred to as NFD-NONcNZO). Mice were received at three to four weeks of age and housed in a barrier facility with a maximum number of two mice caged in standard ventilated mouse cage racks containing recycled heat-treated hardwood Beta chips and cardboard paper bedding (NEPCO, Northeastern Product Corp., NY). Female mice were brought into estrus by cohabiting them in cages containing male bedding for 48 hours prior to mating with males of the same strain. The presence of a vaginal plug the following morning was indicative of successful mating, and these females were considered to be at post-coital and/or gestational day (gd) 0.5. The mice were maintained under a standard 12 hours light/dark photoperiod (light on at 07:00AM) at 20 ± 3 °C with 30%-70% humidity and were allowed free access to water *ad libitum*. Further details on the mice used in this study, husbandry conditions and therapeutic interventions are available in Supplemental Table S1.

### Immune suppression with tacrolimus

According to an established protocol^28^, a modified tacrolimus-based monotherapy was developed for use in the HFD-dNONcNZO mice. Treated obese and diabetic HFD-dNONcNZO mice received tacrolimus (0.1 mg/kg s.c. q2d) (Tacrolimus- Astellas Pharma US, Inc., Deerfield, IL) on an alternate day regimen for four consecutive weeks between HFD weeks 11–14 (corresponding to weeks 15–18 of age) prior to mating (Supplemental Fig. S1). Untreated HFD-dNONcNZO mice and their normative control (NFD-NONcNZO, n = 17) received a mixture of castor oil and ethanol (vehicle for tacrolimus) (Supplemental Table S1). HFD-NONcNZO and NFD-NONcNZO mice were weighed weekly and there had been no signs of drug toxicity such as significant weight loss, tremors or diarrhea.

### Metformin test article and dose formulation

Due to its generic use in the clinical management of individuals with PCOS and to generate a syngeneic control cohort comparing the therapeutic efficacy of tacrolimus in a murine model of PCOS, the broadly prescribed antidiabetic medication metformin was used in the HFD-dNONcNZO mice (Supplemental Table S1 and Fig. S1). Metformin Hydrochloride (C4H_12_CIN_5_, Catalogue #M258815, Toronto Research Chemicals, Toronto, ON) was prepared fresh daily and made available to the HFD-dNONcNZO mice *ad libitum* (200 mg/dL) per day from age weeks 15 to 20 according to an established protocol^28^.

### Collection of ovaries and counting of ovarian structures

#### Collection of ovaries

Through a laparotomy incision, ovaries were collected from anesthetized mated female mice, respectively, at gd 2.5, 4.5 and 6.5, rinsed with ice-cold PBS and then either snapped-frozen in liquid nitrogen or fixed in 4% paraformaldehyde (PFA) for 2 hrs at room temperature.

#### Counting ovarian structures

Using a Leica CM1900 Cryostat and a standard H&E staining protocol^38^, histological ovarian sections were made available for quantifying ovarian follicles, corpora lutea and cysts using a modified validated protocol combining systemic random sampling and the optical dissector method on Image J NIH software system (National Institutes of Health, Bethesda, MD, USA)^39,40^. Briefly, ovaries were sectioned at a 70-µm thickness perpendicular to their longitudinal axes and every 3rd section (5-µm/section) was collected in an order generated on glass slides for staining. This allowed sectioning at the largest two-dimensional profile of the ovaries, and an average of 45 serial sections (5-µm/section) was obtained per ovary. To reduce labor and avoid double counting, 12 slides containing serial ovarian sections were examined by systematically selecting every 4th slide starting at slide 1. Slides were imaged using an x10 oil immersion objective with a high numerical aperture (N^A^ = 1.4) on an Olympus bright-field microscope. Ovarian follicular counts were made by generating an unbiased dissector counting frame with sampling area measuring 6400 µm^2^ on ImageJ^39^. Clearly visible nuclei of oocytes were equated to follicular number and were counted if they were within the unbiased counting frame and not intersected by the exclusion boundaries^39,40^. This allowed a minimum range of 8–12 ovarian follicles from each developmental stage including primordial, primary, secondary, antral and atretic follicles to be identified in individual tissue sections based on the criteria outlined in Myers *et al*.^39^. The mean follicular count was determined for the untreated condition and four weeks of tacrolimus or metformin treatment by averaging the calculated follicular count of each set of serial sections. Corpora lutea were identified as large, round or irregular glandular structures composed of multiple layers of large granulosa lutein cells showing an acidophilic cytoplasm and visible theca lutein cells arranged in clusters. Ovarian cysts were identified as cyst-like structures within the ovary lined by a thin layer of degenerating granulosa cells.

### Confocal microscopy

For identification of the infiltration pattern of M1 ovarian macrophages, fresh-frozen 5 µm thick sections from control, treated and untreated ovaries at gd2.5, gd4.5 and gd6.5 were prepared using a Leica CM1900 Cryostat and placed immediately in 70% ethanol. Slides were hydrated in descending concentrations of ethanol and equilibrated in two changes of PBS prior to applying blocking solution (5% FCS in PBS) for 30 minutes in a humidified chamber at 37 °C. For the identification of the M1 (F4/80+ CD11c+) ovarian macrophages, slides were rinsed with one change of PBS prior to incubation overnight with fluorescent-conjugated primary FITC anti-mouse F4/80 (123107, BioLegend), PE/Cy7 anti-mouse CD11c antibody (117317, BioLegend) or an iso-FITC Rat IgG2a κ (400506, BioLegend) or iso-PE/Cy7 Rat IgG2a κ isotype control antibodies (40092, BioLegend) in a humidified chamber at 4 °C. Similarly, for the immunostaining of the M2 (F4/80+ CD206+) ovarian macrophages, PFA –fixed ovarian sections were incubated overnight in a humidified chamber at 4 °C with fluorescent-conjugated primary FITC anti-mouse F4/80 (123107, BioLegend), phycoerythrin-conjugated anti-mouse CD206 antibody (SC-376108, Santa Cruz Biotechnology), or an iso-FITC Rat IgG2a κ (400506, BioLegend) or phycoerythrin-conjugated goat anti-mouse IgG (ab97024, Abcam Inc.). Subsequently, nuclei were counter-stained with 4’,6-diamidino-2-phenylindole (DAPI) and slides were rinsed twice in PBS and dehydrated in ascending concentrations of ethanol, immersed in three changes of Hemo-D (Fisher Scientific), and mounted using Permount mounting medium (Fisher Scientific). Expression of the F4/80+ CD11c^+^ and the F4/80+ CD206+ cells was observed using a Leica confocal microscope and a Zeiss Axiovert S100 epifluorescent microscope with a Cooke Sensicam high performance camera.

### Flow cytometry: sample preparation and analysis

#### Preparation of peripheral blood mono-nuclear cells (PBMCs)/lymphocytes

An average of 1 × 10^5^ PBMCs was extracted from 0.7–1.0 ml of blood samples collected via cardiac puncture during animal sacrifice using a 27-gauge needle and a syringe containing 300 μl of RBC lysis buffer (pH 7.4) (00-4333-57, eBiosciences, San Diego, CA) and K2EDTA-coated blood collection tubes (BD Vacutainer® Blood collection, Mississauga, Canada). For the rapid recovery of viable lymphocytes from collected blood samples, blood mononuclear cells were separated using Histopaque® - 10831 (Sigma-Aldrich), rinsed twice in a cell staining buffer (420201, BioLegend) containing the viability dye 7-aminoactinomycin D (7-AAD, 00-6993-50, eBioscience) and fixed for 20 minutes at 37 °C in 0.5 ml of a fixation buffer (420801, BioLegend). Isolated lymphocytes were then permeabilized and resuspended in an intracellular staining and permeabilization wash buffer (421002, BioLegend) according to the manufacturer’s instructions. For the identification of the CD8α^+^, CD4^+^, CD25^+^ CD127^low^ Tregs, resuspended lymphocytes were incubated for 45 minutes at 4 °C in dark with the optimum dilution of the following combination of fluorophore-conjugated primary antibodies: anti-mouse CD8α Brilliant Violet 785™ (100749, BioLegend), anti-mouse CD4 APC (100411, BioLegend), anti-mouse CD25 PE/Cy7 (552880, BD Biosciences, South San Francisco, CA, USA) and anti-mouse CD127 PE (557938, BD Biosciences, or their respective IgG2a к isotype controls according to the manufacturer’s suggestions. A BD Pharmingen™ Mouse Th1/Th2/Th17 Phenotyping Kit including CD4 PerCP-Cy5.5, IFNγ FITC, IL-17A PE, IL-4 APC (560758, BD Bioscience Inc., Becton, Dickinson and Company) was used for the identification of the Th1 (CD4^+^ IFNγ^+^), Th2 (CD4^+^ IL4^+^) and Th17 (CD4^+^ IL17A^+^) Tregs following the provider’s recommendations. Mouse IgG1к isotype controls were used to confirm antibody specificity (Supplemental Table S2). Gating strategies and summary of the gating tree for isolated peripheral blood lymphocytes are presented in Supplemental Fig. S2 of the Supplemental Materials and Methods.

#### Preparation of ovarian macrophages

To determine the activation status of ovarian macrophages, an average of 5 × 10^5^ single cell suspensions were generated from ovaries from the NFD-NONcNZO mice, untreated HFD-dNONcNZO and those receiving tacrolimus or metformin on gd 2.5, gd 4.5 and gd 6.5 (n = 10 ovaries/gd/group) by gently purging the ovaries through a 100 µm cell strainer (BD Falcon, San Jose, CA, USA) in RPMI 1640 (Gibco™, Gaithersburg, MD, USA) containing type-IV collagenase, hyaluronidase, and DNase I (all from Sigma, St Louis, MO, USA). Erythrocytes lysed in ovarian suspensions using an RBC lysis buffer (pH 7.4) (00-4333-57, eBiosciences) for one minute each at 37 °C, and leukocytes were isolated by percoll density gradient centrifugation (40% and 80%, 320 × g for 25 minutes at 37 °C) (Sigma-Aldrich, St Louis, MD, USA) according to the manufacturer’s instructions and following a modified standard protocol^41^. Isolated ovarian leukocytes were washed twice in ice-cold PBS before being re-suspended for 10 minutes on ice in 0.1 ml of fixation buffer containing 3% PFA (420801, BioLegend) and viability dye 7-AAD (00-6993-50, eBioscience). Fixed cell suspensions were washed twice in ice-cold PBS, and re-suspended in permeabilization and blocking buffer (421002, BioLegend) containing 10% fetal calf serum (FCS) for 20 minutes on ice. The samples were incubated with a panel of fluorescence-conjugated antibodies (Supplemental Table S2) for 30 minutes on ice in an incubation buffer containing 5% FCS. Labeled cell suspensions were rinsed twice in 0.5 ml permeabilization wash buffer (421002, BioLegend) and re-suspended in 0.5 ml ice-cold PBS containing 5% FCS (Thermo-Fisher Scientific, Mississauga, Ontario, Canada). Fluorescent activated cell sorting and immunophenotyping were performed on a Beckman Coulter (BC) FC500 flow cytometer (Beckman Coulter, Ontario, Canada) using Summit software 4.3. Post-acquisition data analysis was performed using FlowJo™ v 10.4.2. Summary of gating for isolated ovarian macrophages are presented in Supplemental Fig. S3 of the Supplemental Materials and Methods.

### Cytokine proteome profiler array analysis

Quantification of ovarian and serum levels of cytokines in the HFD-dNONcNZO dams and those receiving metformin (200 mg/dL) or tacrolimus (0.1 mg/Kg) relative to their normative control mice (NFD-NONcNZO) was performed using the Mouse Cytokine Proteome Profiler Array Panel A kit (R&D Systems) according to the manufacturer’s exact specifications. For maximum sensitivity, supernatants (500 μl each) of samples were incubated with the supplied Cytokine Array Panel A Antibody Cocktail for 24 hours at 4 °C under constant agitation. The array membranes were blocked with the supplied blocking buffers prior to incubating them with the supernatant-antibody mixtures overnight at 4 °C on a shaking platform. Array membranes were then washed in wash buffers and were incubated with streptavidin-HRP [1:500 (vol/vol) in 5% (vol/vol) non-fat milk (Thermo-Fisher Scientific, Canada) in PBS-T] for 30 minutes at 23 °C. Enhanced chemiluminescence was conducted for 1 and 3 minutes using the SuperSignal West Femto chemiluminescent kit (Thermo-Fisher Scientific, Canada). Images were then captured and analyzed using Alpha-Innotech H2D Imager coupled with AlphaEase FC software (version 4.1, Alpha Innotech, San Leandro, CA). Fold changes in ovarian and serum cytokine and chemokine expressions for the 1 and 3 minutes exposures were calculated using the equation “Fc = log2 (Eka) − log2 (Ekb)” where ‘Eka’ is the expression level of the control dams and Ekb is that of the test group. A 1.5 fold change in the averaged cytokine and chemokine expression was considered significant (*p* < 0.01) at 95% confidence.

### Statistical analysis

Data were analyzed with Sigmaplot (Systat Software, Inc., San Jose, CA) and Graph-Pad Prism 6 software. Normal distributions of data were confirmed using the Kolmogorov-Smirnov test. Two-way analysis of variance (ANOVA) comparing treatment groups with their untreated and normative control mice was also used to determine alpha (*p*) values for the source of variation across treatments. *t* and *p* values for the statistical differences among mean immune cells values across experimental mouse groups were determined by One way ANOVA followed by Student’s *t-*test. Linear (parametric) data were assessed by Pearson correlation, whereas nonlinear (non-parametric) data were assessed by Spearman correlation. Parameters of normally distributed data were expressed, unless otherwise indicated, as mean ± SEM, mean ± SDM or range ± ME using appropriate non-parametric procedures (Fisher Exact test; Kruskal–Wallis and/or one-way ANOVA with interaction effects) followed by Dunn’s multiple comparison test or Mann-Whitney U test or Miller’s procedure for pairwise comparisons of independent parameters.

### Ethical approval

All experimental procedures, methods and animal usage reported in the present investigation were in accordance with the ethical guidelines mandated by the Canadian Council on Animal Care and approved by the Animal Care Committee of Queen’s University, Kingston, Ontario Canada (protocol # Kan-OR-013).

## Results

### Alterations in the local ovarian milieu and the effect of tacrolimus in the HFD-dNONcNZO mice

#### Ovarian morphology and analysis of ovarian follicles

**Figure 1:**
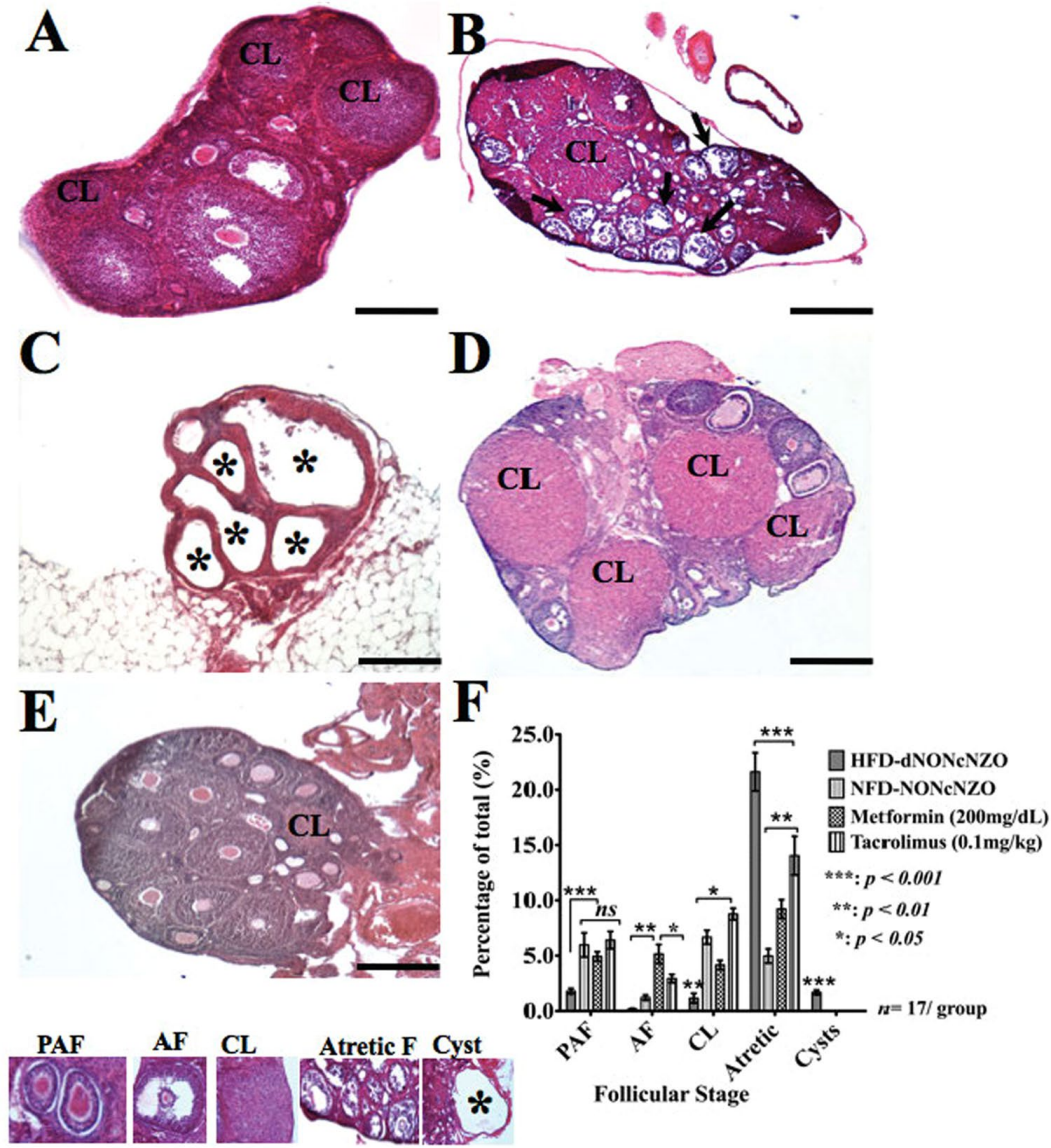
Immunosuppression with tacrolimus supported functional ovarian phenotype in the HFD-dNONcNZO mice. Representative H&E stained ovarian sections from NFD-NONcNZO (**A**), HFD-dNONcNZO (**B**,**C**), tacrolimus- (**D**) and metformin- (**E**) treated mice. As opposed to normal ovarian morphology in the NFD-NONcNZO mice (**A**), follicular atresia (**B**: arrows) and large follicular cysts (**C**: *) characterized ovaries obtained from untreated diabetic HFD-dNONcNZO mice. Despite significant differences among treated mice (compare *p* values among treated mice at 95% confidence), treatment with tacrolimus (**D**) or metformin (**E**) inhibited ovarian cyst formation, suppressed follicular atresia and supported ovulatory phenotypes as judged by the development of antral follicles and persistence of post-ovulatory corpora lutea (CL) at gd4.5 in treated mice. (**F**) histogram comparing frequency of distribution of various ovarian structures presented in (**A**–**E**). Although treatment with tacrolimus or metformin supported the growth of pre-antral follicles (PAF) in treated mice, the use of tacrolimus significantly inhibited premature luteolysis as judged by the significant presence of post-ovulatory corpora lutea in treated mice (mean difference between treatment groups = 2.51, *p* = 0.012, t = 3.361; 95% confidence interval = 0.391–4.619). An average of 45 serial sections (5 µm/section, 320 µm of tissue) was obtained per ovary. At least 4–5 animals with 12 ovarian sections per animal were used in each experiment. 8–12 ovarian structures including ovarian follicles, cysts and corpora lutea were analyzed per mouse per group. Scale bar = 200 μm in (**A**–**E**). PAF: Pre-antral Follicle; AF: Antral Follicle, CL: Corpus Luteum, Atretic F: Atretic Follicle.

Ovarian sections stained with H&E revealed marked differences between the control (Fig. 1A), untreated (Fig. 1B,C) and treated HFD-dNONcNZO mice (Fig. 1D,E) in terms of ovarian morphology and folliculogenesis. Ovaries of untreated HFD-dNONcNZO mice have higher percentages of atretic follicles (Fig. 1B) and ovarian cysts (Fig. 1C) compared to corresponding diabetic HFD-dNONcNZO mice treated either with tacrolimus (Fig. 1D) or metformin (Fig. 1E; see also bar graphs in Fig. 1F for a comparison). Consistency in counting ovarian follicles among all experimental groups of mice included in this study was a major challenge. Great care was taken in identifying various ovarian follicles by virtue of their histological characteristics. These include counting every primordial follicle, as well as every growing follicle with the nucleus of the oocyte clearly visible according to an established protocol^42^. Clear patterns were observed in the types of ovarian follicles present in treated and untreated diabetic HFD-dNONcNZO mice. Untreated HFD-dNONcNZO mice developed intra-ovarian cysts with a mean ± SEM diameter of 2.13 ± 0.19 mm ranging from 1.24–4.37 mm in diameter These ovarian cysts were morphologically distinct from antral follicles which measured a range of 0.48–1.23 mm in diameter (with a mean ± SDM value of 0.59 ± 0.046) (Supplemental Table S3). Treated diabetic HFD-dNONcNZO mice showed significant abundance of corpora lutea and developed ovarian follicles at the pre-antral and antral stages (Fig. 1D,E, and bar graphs in Fig. 1F**)** reflecting a beneficial effect of tacrolimus on folliculogenesis.

#### Activation profile and localization of ovarian macrophages

**Figure 2:**
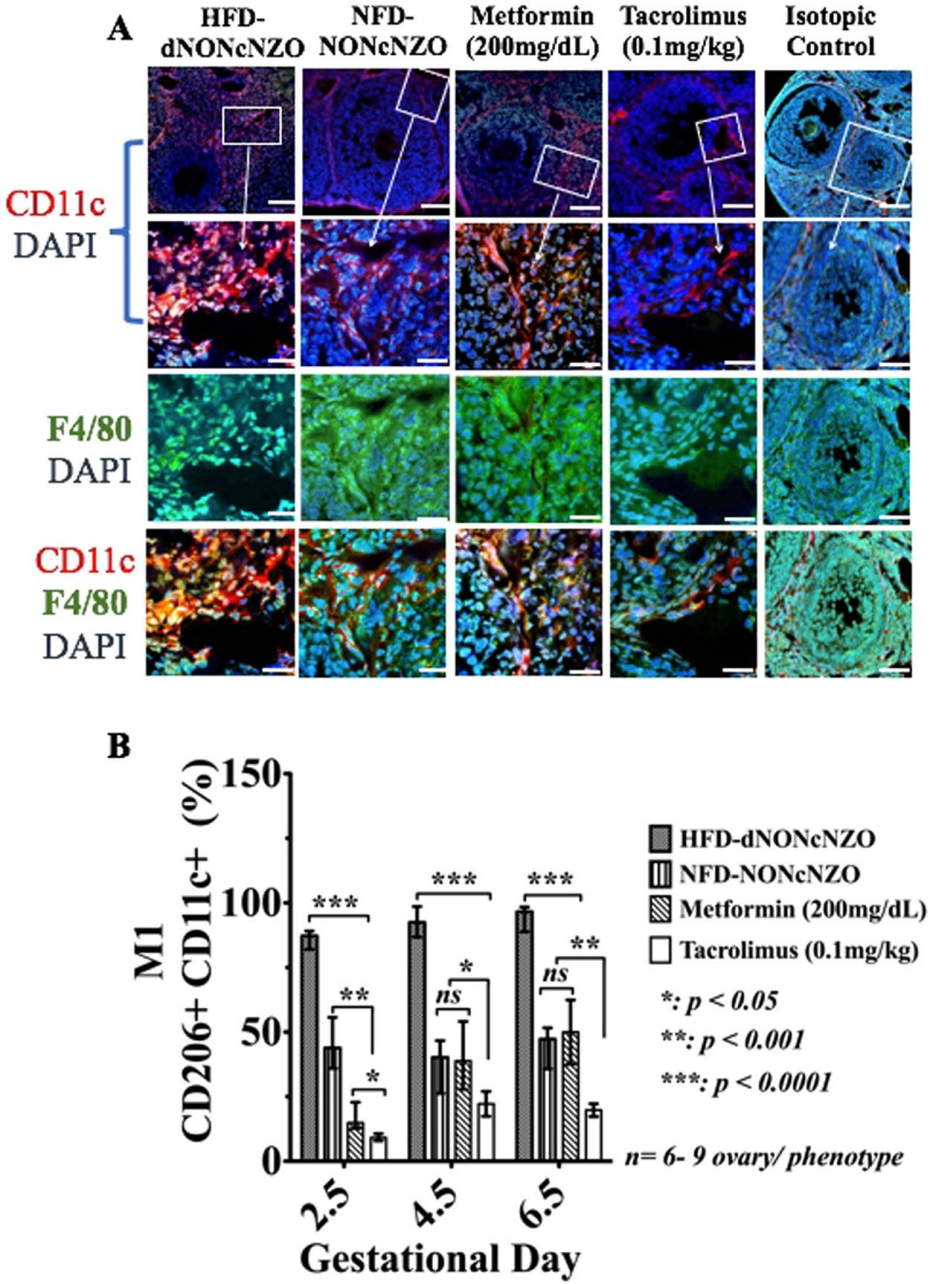
Mono-therapeutic interventions with tacrolimus suppressed aberrantly activated ovarian F4/80^+^ CD11c^+^ macrophages in treated mice. Detection of M1 activated F4/80^+^ CD11c^+^ macrophages in the tacrolimus- and metformin-treated mice and their untreated and control groups at postcoital/gd 4.5 by confocal microscope (**A**) and by flow-cytometry (**B**). Red fluorescence in the upper two horizontal panels in (**A**) indicates the presence of CD11c^+^ and the green fluorescence in the third horizontal panel is the surface staining for F4/80^+^ macrophages, respectively. The merged fluorescence images in the lower horizontal panel in (**A**) (orange) further identify the classically activated F4/80^+^ CD11c^+^ macrophages. As opposed to the NFD-NONcNZO and those receiving tacrolimus or metformin, infiltration of the vascular granulosa cells layer with CD11c^+^ macrophages was pathognomonic feature of uncoordinated follicular growth among untreated HFD-dNONcNZO mice. Figures in the second horizontal panel in **A** are high magnifications of the corresponding framed boxes shown in the first horizontal panel. Bars in **A** = 100 μm. The peri-conceptional differential expression profiles of the activated ovarian M1 (CD11c^+^ CD206^+^) macrophages during postcoital days 2.5, 4.5 and 6.5 in all experimental groups are represented in bar graphs in (**B**). Nuclei (blue fluorescence) were counterstained with DAPI.

**Figure 3:**
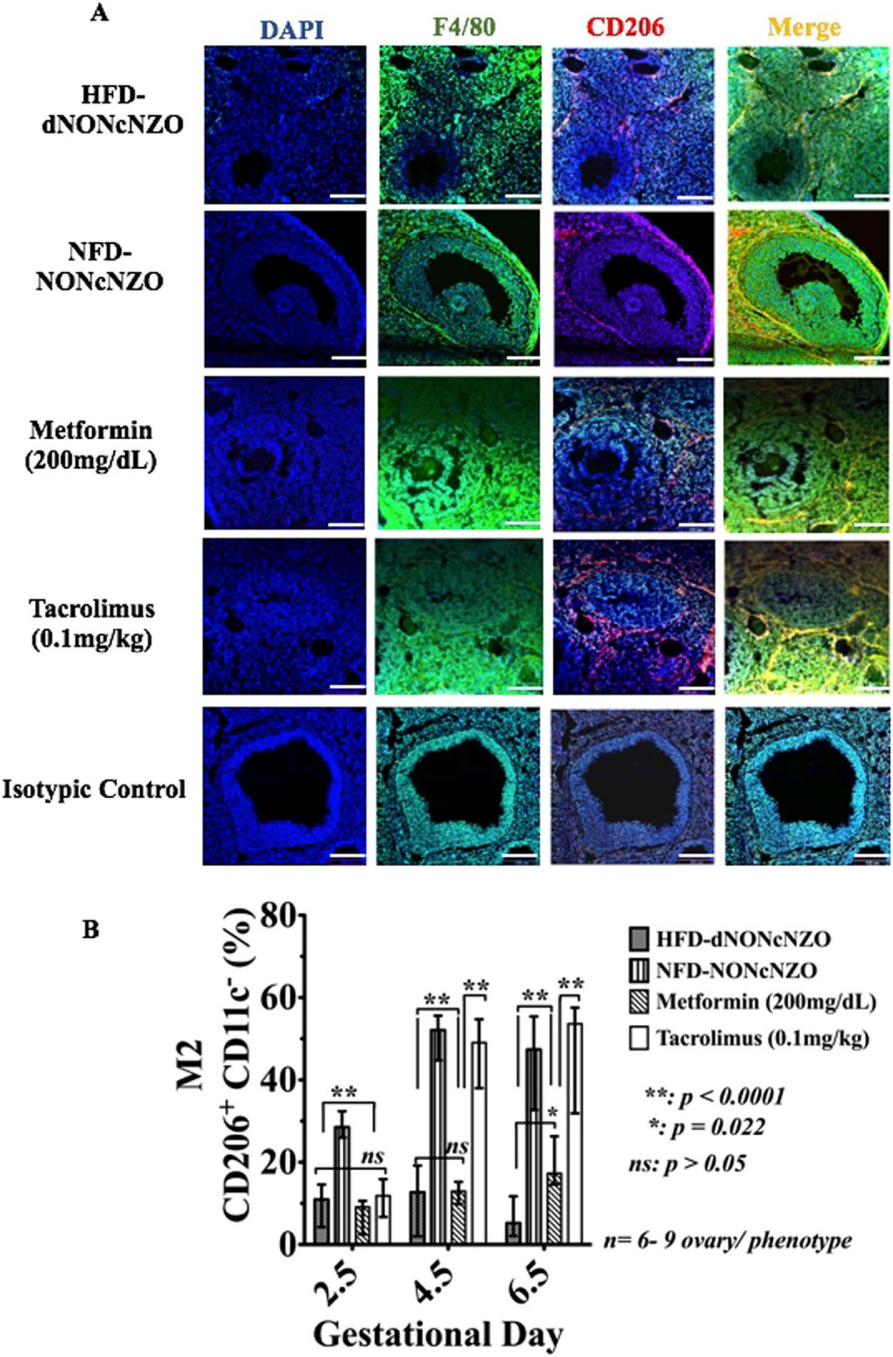
The systemic use of tacrolimus (0.1 mg/kg) for four consecutive weeks influenced histological distribution and activation profile of ovarian F4/80^+^ CD206^+^ macrophages in treated mice. Shown is the detection of M2 activated F4/80^+^ CD206^+^ ovarian macrophages in histological sections (**A**) and by flow-cytometry (**B**) (CD206^+^ CD11c^−^) in the tacrolimus- and metformin-treated mice and their untreated and control groups at postcoital/gd 4.5. Red fluorescence in (**A**) indicates the presence of CD206^+^ macrophage**s**, and the green fluorescence is the surface staining for F4/80^+^ cells. The merged fluorescence images in (**B**) (yellow) further identify the alternatively activated F4/80^+^ CD206^+^ ovarian macrophages, respectively. Bar graphs in (**B**) represent the peri-conceptional differential expression profiles of the M2 (CD206^+^ CD11c^−^) activated ovarian macrophages during postcoital days 2.5, 4.5 and 6.5 in all experimental groups. As opposed to the NFD-NONcNZO and those receiving tacrolimus, low-level infiltration of the vascular granulosa cells layer with F4/80^+^CD206^+^ macrophages was the hallmark of perturbed follicular growth among untreated HFD-dNONcNZO mice. Notably, therapeutic intervention with metformin (200 mg/dL) failed in promoting the peri-conceptional expansion of the CD206+CD11c− macrophages in treated mice (compare merged fluorescence in (**A**) and bar-graphs in (**B**) in the metformin-treated mice to those receiving tacrolimus in (**A**,**B**), respectively). Bars in **A** = 100 μm. Nuclei (blue fluorescence) were counterstained with DAPI.

Among prominent features of a perturbed peri-conceptional ovarian milieu in the HFD-dNONcNZO mice were the aberrant over-expression and altered tissue distribution of the classically activated ovarian M1 (F4/80^+^ CD206^+^ CD11c^+^) macrophage cell populations as well as restricted expansion of the M2 (F4/80^+^ CD206^+^ CD11c^−^) cells at gd 2-5- gd 6.5, respectively. As shown in Figs 2A,B, 3A,B as well as Supplemental Figs S4A–D and Ai- Di, comparative histological and cellular ovarian studies revealed excessive ovarian infiltration with F4/80^+^ CD206^+^CD11c^+^ macrophages and limited peri-conceptional expression of the F4/80^+^CD206^+^CD11c^−^ ovarian macrophages in the HFD-dNONcNZO mice at gd 2.5- gd 6.5 respectively. Major differences emerged comparing the immunosuppressive effect of tacrolimus (0.1 mg/kg) to that of the monotherapeutic intervention with metformin (200 mg/dL) on the peri-conceptional homeostasis of the ovarian M1 and M2 macrophages. As depicted in Figs 2A,B, 3A,B and Supplemental Figs S4A–D and Ai- Di, the use of tacrolimus rather than metformin significantly (*p* < 0.01) restored the cellular expression and histological distribution of the M2 while further inhibiting aberrantly expressed M1 ovarian macrophages. The average percentages of ovarian F4/80^+^ CD206^+^ CD11c^+^ and F4/80^+^ CD206^+^ CD11c^−^ cells were calculated in all experimental groups at postcoital/gestational days 2.5, 4.5 and 6.5, respectively (Supplemental Table S4). Therefore it is evident that the use of low dose tacrolimus (0.1 mg/kg) in conditions of chronic maternal overnutrition supports peri-conceptional expansion of alternatively activated ovarian M2 macrophages in the obese and diabetic HDF-dNONcNZO mice.

#### Ovarian cytokines profile at gd 4.5

**Figure 4:**
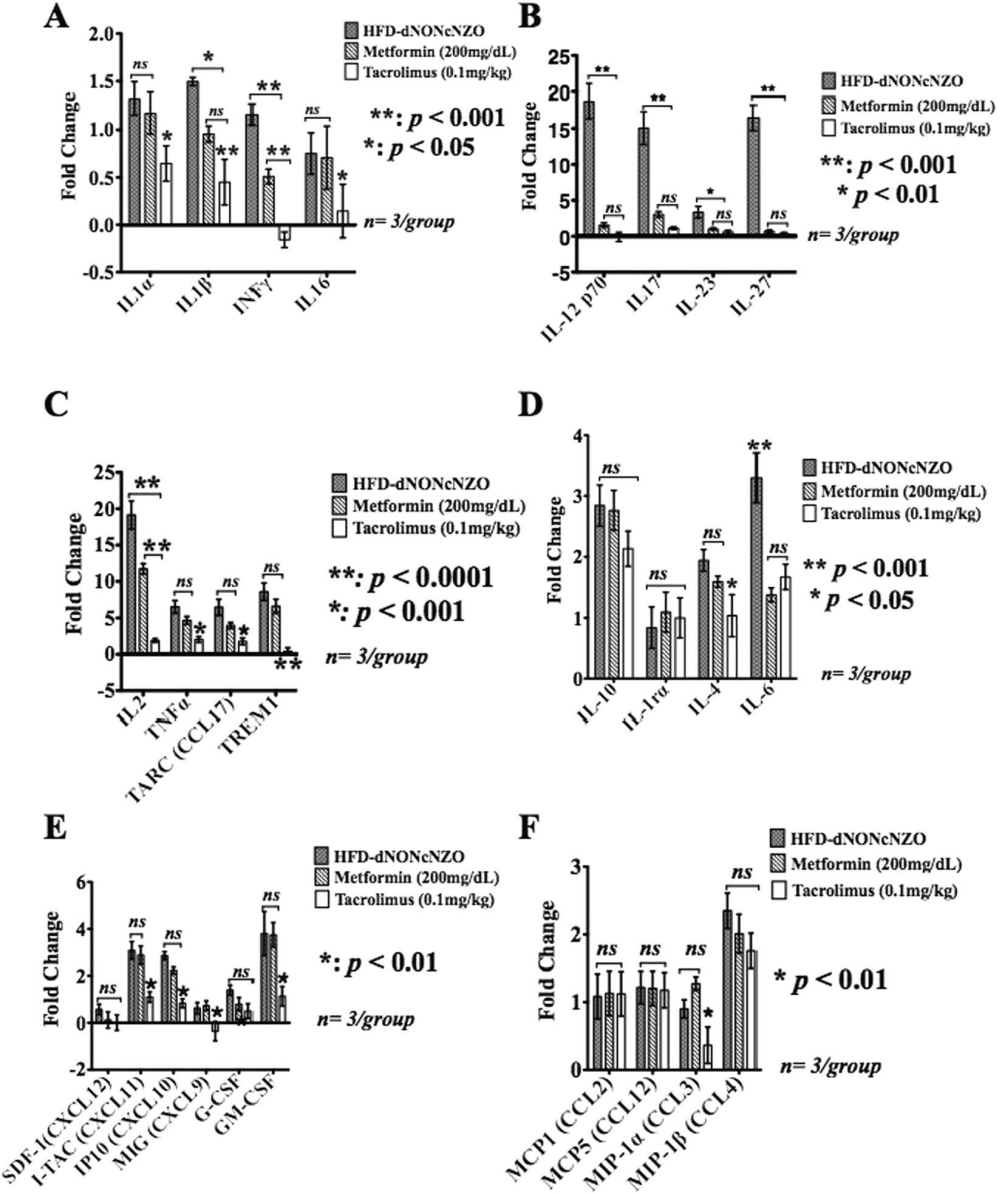
Immunosuppression with tacrolimus inhibited an adversary pro-inflammatory ovarian milieu in the HFD-dNONcNZO mice. Using a membrane-based antibody array for the parallel determination of the relative levels of selected mouse ovarian cytokines and chemokines, bar graphs in **A**–**F** are representations of mean ± SDM of three replicates measuring the expression of ovarian cytokines and chemokine receptors and their ligands at postcoital/gd 4.5 among mated untreated and treated HFD-dNONcNZO mice. A 1.5-fold change in the protein expression of the cytokine relative to that of the NFD-NONcNZO mice was considered significant (*p* < 0.01 at 95% confidence, n = 3/phenotype). Of the most upregulated peri-conceptional ovarian cytokines and chemokines in the HFD-dNONcNZO mice which were significantly inhibited using tacrolimus were the pro-inflammatory mediators IL1β and IFNγ (**A**), IL12p70, IL17, IL23 and IL27 (**B**), IL2, TNFα and its downstream targets TREM1 (Triggering Receptor Expressed On Myeloid Cells 1) and TARC (CCL17: Thymus and Activation Regulated Cytokine) (**C**), IL6 (**D**), GM-CSF and the CXC ligands 9, 10 and 11 (**E**) and the monocytes-macrophage regulatory chemokine MIP-1α (CCL3) (**F**). Except for suppressing IL6 (*p* < 0.01) and a significant trend in downregulating IL4 by use of tacrolimus (mean difference between treated and untreated = 0.91, *p* = 0.031, t = 2.798; 95% confidence interval = 0.027–1.786), there has been no significant downregulation of the anti-inflammatory cytokines IL10 and IL1r α (*p* > 0.05) (**D**). Constitutively, no significant pan-cytokine suppression was observed in the metformin-treated mice apart from a significant downregulation of IFNγ (**A**), IL12/IL17 family members (**B**), IL2 (**C**) and IL6 (**D**). Due to the gestational age examined and the low dose of tacrolimus (0.1 mg/kg), the monocytes and macrophage chemoattractant and regulatory chemokine ligands MCP1 (CCL2), MCP5 (CCL12) and MIP-1β (CCL4) (**F**) were the least affected chemokines in the tacrolimus- and the metformin-treated mice.

Consistent with the impact of HFD in provoking a pro-inflammatory ovarian milieu in mice^9^, and the cytokine-modulating effects of tacrolimus^43^, we analyzed 37 of the murine cytokines and chemokine ligands and their receptors which are known to influence ovarian functions in PCOS^44^. Compared to those receiving metformin and their respective control mice, our data revealed a differential suppressive effect of tacrolimus on certain pro-inflammatory and anti-inflammatory cytokines and chemokines during early pregnancy in the HFD-dNONcNZO mice. As shown in Fig. 4, of the most significantly (i.e. a fold-change of 1.5 times compared to control NFD-NONcNZO) upregulated peri-conceptional ovarian cytokines, chemokine receptors and their ligands which were significantly inhibited (*p* < 0.01) by use of tacrolimus were IFNγ (Fig. 4A), IL12p70, IL17 and IL27 (Fig. 4B), IL2, TNFα, TARC (CCL17) and TREM1 (Fig. 4C), IL6 (Fig. 4D), the CXC-motif ligands CXCL9, 10 and 11 and GM-CSF (Fig. 4E) as well as the monocytes inhibitory protein 1α (MIP-1α/CCL3) (Fig. 4F). Except for a moderate (*p* < 0.05) suppressive effect on IL4 (Fig. 4D), we did not detect a significant (*p < *0.01) suppressive effect of tacrolimus on the anti-inflammatory cytokines IL10 and IL1rα (Fig. 4D), neither were the monocytes regulatory proteins MCP1/CCL2, MIP-1β/CCL4 and MCP5/CCL12 (Fig. 4F) affected by the current therapeutic interventions with tacrolimus.

### Peri-conceptional alterations in the systemic immune niche and effect of tacrolimus on the HFD-dNONcNZO mice

#### HFD-dNONcNZO mice expressed low levels of circulating CD4^+^ and CD8α^+^ lymphocytes and diminished peri-conceptional expansion of the CD25^+^CD127low T cells

**Figure 5:**
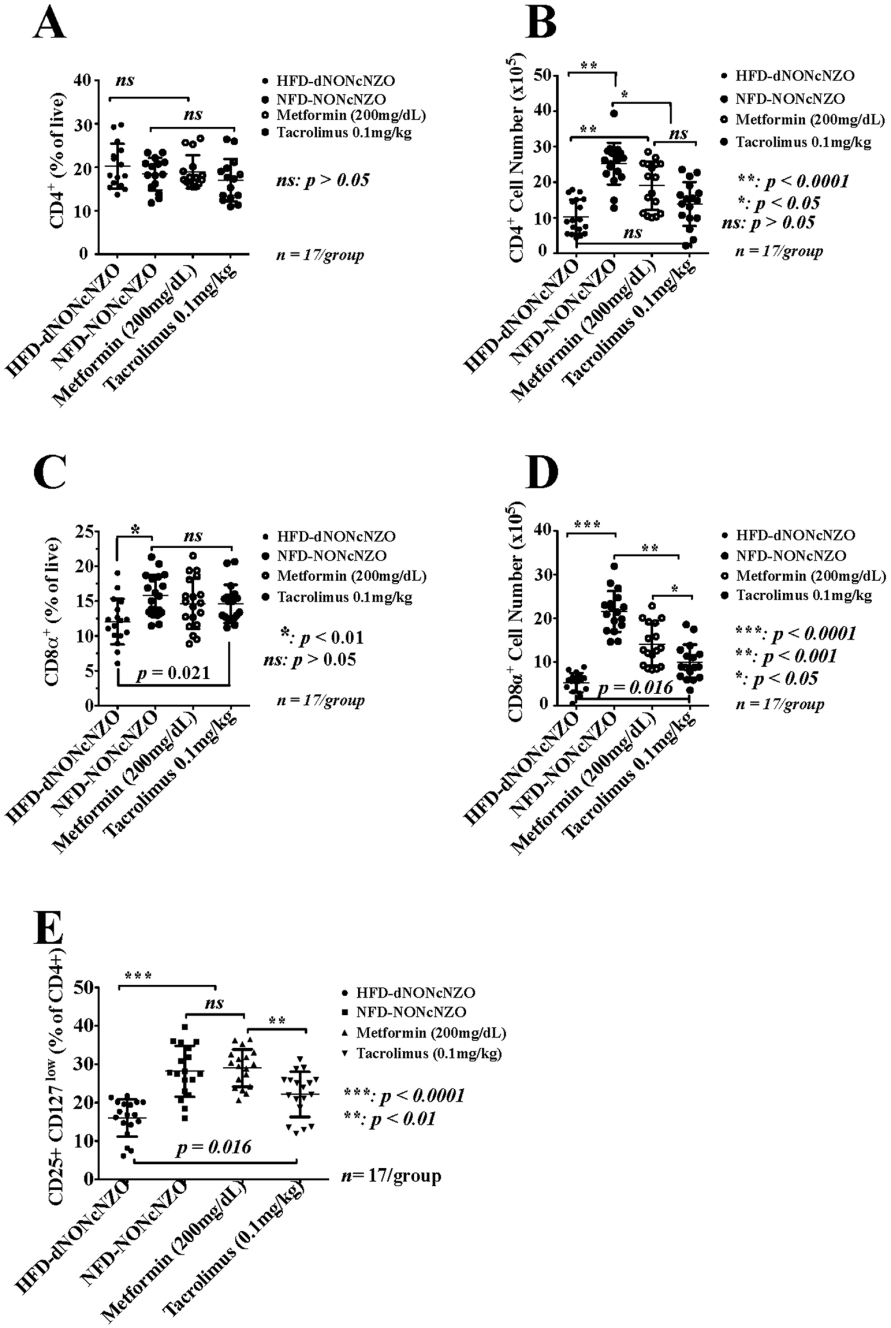
The peri-conceptional status of circulating CD4^+^, CD8α^+^ and CD25^+^CD127^low^ lymphocytes in treated HFD-NONcNZO dams. Flow-cytometric analysis of proportions and numbers of peripheral CD4^+^ T cells (**A**,**B**) and CD8α^+^ T cells (**C**,**D**) as well as CD25^+^CD127^low^ T cells (**E**) revealed reduced total leukocytes and impaired peri-conceptional expansion of peripheral CD4^+^ CD25^+^ CD127^law^ and CD8α^+^ T cells in untreated HFD-dNONcNZO mice. A mean difference in the value of (-14961) (*p* < 0.0001, t = 7.569; 95% confidence interval = −20631–−9.290) in the numbers of circulating CD4^+^ cells between the untreated HFD-dNONcNZO (mean ± SDM = 1029 ± 481 *vs* control NFD-NONcNZO mice: 25252 ± 5862) and those receiving metformin (19045 ± 6760) (mean difference = −8754, *p* < 0.001, t = 4.365; 95% confidence interval = 14507–−3000) was calculated. Although no significant difference in the proportions and numbers of circulating CD4^+^ T cells was observed between the tacrolimus-treated *vs* untreated HFD-dNONcNZO mice (mean difference = −3611, *p* = 0.363, t = 1.8008; 95% confidence interval = −9365– 2142), a significant increase in the proportions and numbers of CD8α^+^ (**C**: *p* < 0.01 and **D**: *p* < 0.05) and CD25^+^ CD127^low^ T cells (**E**: *p* = 0.016) was detected among treated mice. Proportions of CD25^+^ CD127^low^ cells were quantified after gating on a CD4^+^ channel which allowed for the clear separation of the CD4^+^ CD25^+^CD127^low^ Tregs niche (Supplemental Fig. S2D). Antibody specificity was confirmed using isotypic controls and histograms representing mean fluorescence intensities (MFI) of CD4^+^, CD8α^+^ and CD25^+^CD127^low^ T cells were generated (Supplemental Fig. S2C,D).

Due to the regulatory role of maternal CD4^+^ and CD8α+ lymphocytes in establishing maternal tolerance during early pregnancy and in congruity with previous reports on the defective expansion of the CD4^+^ CD25^+^ CD127^low^ cells at the follicular phase of the menstrual cycle in women with PCOS^7,45^, proportions and absolute numbers of circulating CD4^+^, CD8α^+^ and CD25^+^CD127^low^ in the HFD-dNONcNZO mice were analyzed at the day of implantation (gd4.5). We tested a null hypothesis that the obese and diabetic HFD-dNONcNZO mice with PCOS suffer from Tregs expansion defects during early pregnancy. As demonstrated in Fig. 5(A–E), despite comparable proportions of the CD4^+^ lymphocytes analyzed in all experimental groups (Fig. 5A), untreated HFD-dNONcNZO mice were significantly low (*p* < 0.001) in numbers of peripheral CD4^+^ (Fig. 5B) as well as proportions and numbers CD8α^+^ (Fig. 5C,D) at gd 4.5. Constitutively, low proportions of the CD25^+^CD127^low^ T cells were detected among untreated HFD-dNONcNZO mice (mean ± SDM = 15.97 ± 4.84) compared to control NFD-NONcNZO (28.16 ± 6.62, t = 6.528, *p* < 0.0001) and those receiving tacrolimus (22.15 ± 5.87, t = 3.305, *p* = 0.0168) or metformin (29.01 ± 4.86, t = 6.981, *p* < 0.0001) (Fig. 5E, Supplemental Table S5 and Fig. S5A,B). Due to the pan-suppressive effect of tacrolimus, a significant mean difference in the percentages of CD25^+^CD127^low^ T cells in the value of −6.858 (*p* = 0.0061, t = 3.675) was detected between the tacrolimus- and the metformin-treated mice.

#### HFD-dNONcNZO mice exhibit distinct peri-conceptional perturbations in the phenotypic frequencies of peripheral T helper 1 (CD4+ IFNγ+) and T helper 2 (CD4+IL4+) cells

**Figure 6:**
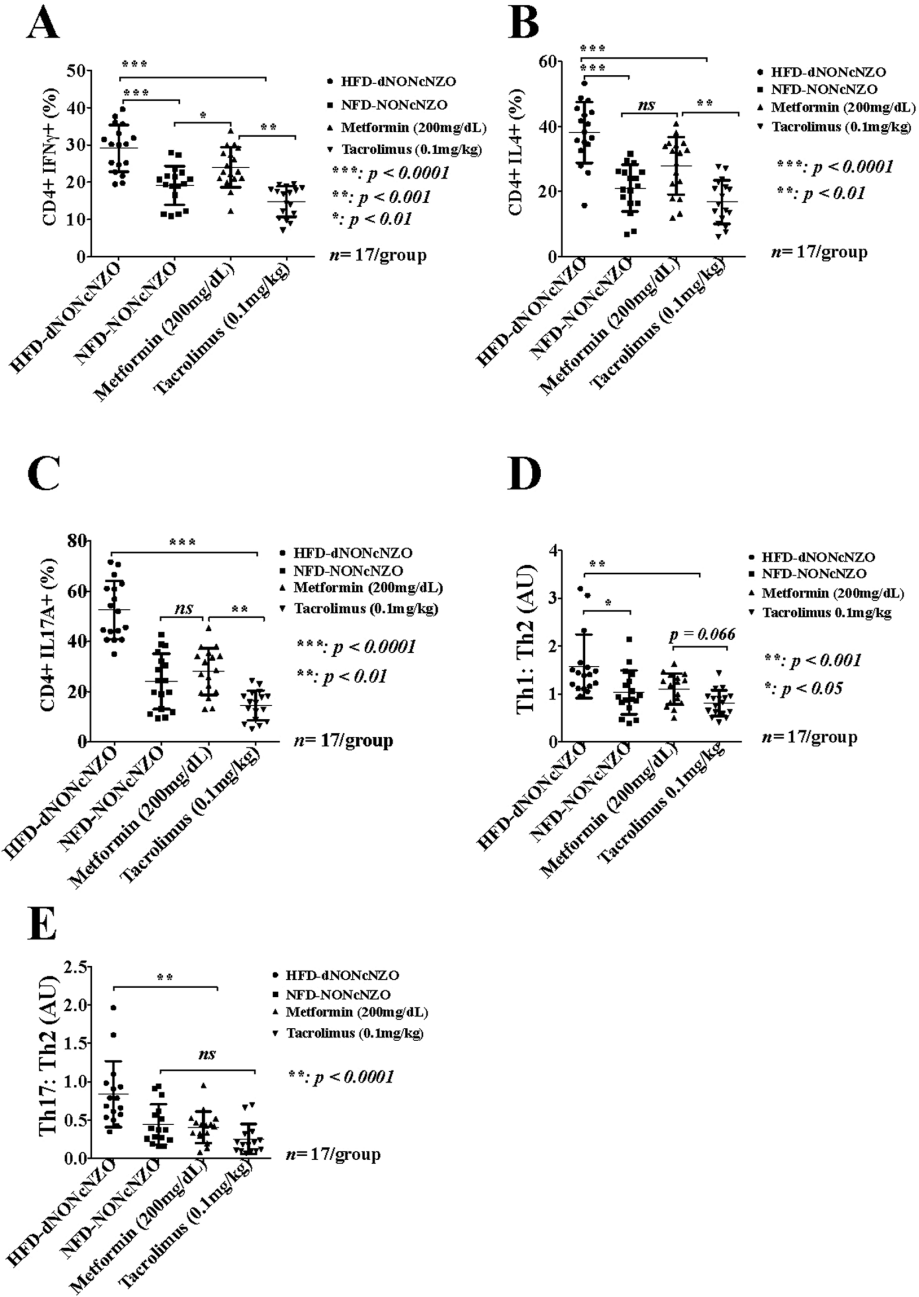
Effect of HFD and immunosuppression with tacrolimus on the peri-conceptional phenotypic frequencies and proportions of circulating maternal CD4^+^IFNγ^+^, CD4^+^IL4^+^ and CD4^+^IL17A^+^ Tregs and alterations to the Th1:Th2 and Th17:Th2 cell ratios in the HFD-dNONcNZO mice. (**A**–**C**) Graphic representations of mean ± SDM of percentages (%) of CD4^+^ T cells gated for their fluorescence activated intracellular staining for IFNγ (**A**), IL4 (**B**) and IL17A (**C**) in lymphocytes of HFD-dNONcNZO mice (n = 17), their normative control NFD-NONcNZO (n = 17) and those receiving metformin (n = 17) or tacrolimus (n = 17). (**D**,**E**) depict, respectively, ratios of circulating Th1 (CD4^+^IFNγ^+^): Th2 (CD4^+^IL4^+^) (**D**) and Th17 (CD4^+^IL17A^+^): Th2 (CD4^+^IL4^+^) (**E**) cells at gd 4.5 among experimental groups. Compared to control values, the Th1:Th2 cell ratio (**D**) was significantly elevated in the untreated HFD-dNONcNZO mice (mean difference = 0.698, *p* < 0.05, t = 7.721; 95% confidence interval = 0.438–0.959) *vs* the tacrolimus- (mean difference = 0.752, *p* < 0.001, t = 8.304; 95% confidence interval = 0.491–1.012) or the metformin-treated HFD-dNONcNZO mice (mean difference = 0.728, *p* < 0.001, t = 8.054; 95% confidence interval = 0.468–0.989). Constitutively, the use of both treatment modalities significantly inhibited an aberrant peri-conceptional Th17:Th2 cell ratio (**E**) in the HFD-dNONcNZO mice (mean difference = 1.147, *p* < 0.0001, t = 5.855; 95% confidence interval = 0.584–1.712 compared to the tacrolimus-treated mice *vs* a mean difference of 1.044, *p* = 0.0001, t = 5.324; 95% confidence interval = 0.479–1.607 in the metformin-treated mice).

Due to the positive effect of tacrolimus on functional CD4^+^ Regulatory T cells^35,46^ and their numerical and functional deficits in individuals with PCOS^7^ we examined the impact of chronic HFD administration and the effect of tacrolimus on the phenotypic expression and absolute numbers of the Th1 (CD4^+^IFNγ^+^) and Th2 (CD4^+^IL4^+^) cells at gd 4.5 in the NONcNZO mice. As shown in Fig. 6A, Supplemental Tables S6 and S7, respectively and scatterplots in Supplemental Figs S6A and S6B, untreated HFD-dNONcNZO mice have significantly elevated numbers of CD4^+^ IFNγ^+^ Th1 (29.11 ± 6.28) compared to control NFD-NONcNZO (19.15 ± 5.19, t = 9.955, *p* < 0.0001) *vs* those receiving tacrolimus (14.85 ± 4.08, t = 7.861, *p* < 0.0001) or metformin (24.04 ± 5.35, t = 2.796, *p* < 0.001). Furthermore, analysis of the CD4^+^ IL4^+^ T cells revealed significantly elevated percentages among the untreated HFD-dNONcNZO mice (38.11 ± 9.33) compared to control NFD-NONcNZO (21.07 ± 7.17, t = 6.144, *p* < 0.0001) *vs* those receiving tacrolimus (16.81± 6.70, t = 7.683, *p* < 0.0001) or metformin (27.89 ± 8.81, t = 3.687, *p* < 0.01) (Fig. 6B and Supplemental Table S7 and scatterplots in Supplemental Fig. S6B).

#### The peri-conceptional status of the T helper 17 (CD4^+^ IL17A^+^) cells in the HFD-dNONcNZO mice

Among the T cell niche examined in this investigation was the Th17 (CD4^+^IL17A^+^) cells and their prototypical secretion of IL17A in the HFD-dNONcNZO mice. As shown in Fig. 6C, Supplemental Table S8 and scatterplots in Supplemental Fig. S6C, untreated HFD-dNONcNZO mice have a substantially elevated percentage of circulating Th17 (CD4^+^ IL17A^+^) cells (52.52 ± 11.60) compared to their normative control (24.11 ± 11.07, t = 8.495, *p* < 0.0001) *vs* the tacrolimus- (14.53 ± 5.92, t = 11.363, *p* < 0.0001) or the metformin-treated mice (28.02 ± 9.36, t = 7.327, *p* < 0.01).

#### The peri-conceptional status of the Th1:Th2 and Th17:Th2 cell ratios in the HFD-dNONcNZO mice

In calculating ratios of Th1:Th2 we found untreated HFD-dNONcNZO mice expressing higher than normal ratios of Th1:Th2 cells (1.58 ± 0.66) compared to normative controls (1.03 ± 0.45, t = 3.044, *p* = 0.033), and those treated with tacrolimus (0.81 ± 0.26, t = 4.294, *p* = 0.0009) (Table 1, Fig. 6D). We have also found that in restoring fertility in the HFD-dNONcNZO mice^29^, the use of metformin was also effective in favorably altering the Th1:Th2 cell ratios among treated mice compared to the untreated (mean difference = 0.473, t = 3.944, *p* < 0.001). A statistical trend (*p* = 0.066) was observed between treatment with tacrolimus *vs* that of metformin (mean difference = 0.495, t = 2.781; 95% confidence interval = −0.0160–1.006). Nonetheless, both treatment modalities significantly inhibited an aberrantly elevated Th17:Th2 cell ratios in the untreated HFD-dNONcNZO mice (0.84 ± 0.43) compared to control values (0.44 ± 0.26, t = 5.644, *p* < 0.0001) *vs* the tacrolimus- (0.25 ± 0.19, t = 5.855, *p* < 0.0001) and the metformin-treated mice (0.41± 0.21, t = 5.323, *p* < 0.0001) (Table 2, Fig. 6E). Thus, compared with metformin, it is evident that parallel with the human studies^46^ the peri-conceptional use of tacrolimus (0.1 mg/kg) is also sufficient to normalize abnormally elevated Th1:Th2 and Th17:Th2 cell ratios conducive to gestational success in obese individuals with PCOS.

**Table 1:** Ratio of circulating Th1 (CD4+IFNγ+): Th2 (CD4+IL4+) cells at gd 4.5 among experimental groups.

	HFD-dNONcNZO	NFD-NONcNZO	Metformin (200 mg/dL)	Tacrolimus (0.1 mg/kg)
^#^Animals	17	17	17	17
Range	0.95–3.20	0.38–2.13	0.50–1.63	0.41–1.43
Median ±ME	1.40 ± 0.05*	0.94 ± 0.03*	1.17 ± 0.02*	0.83 ± 0.02**
Mean ± SDM	1.58 ± 0.66	1.03 ± 0.45	1.11 ± 0.32^†^	0.81 ± 0.26^†^

*p < 0.01 comparing group medians at alpha 0.05 (One-way ANOVA followed by Kruskal-Wallis test).

**p < 0.001 comparing maximal differences between the tacrolimus-treated *vs* untreated HFD-dNONcNZO group medians at 95% confidence interval (Mann-Whitney U test).

^†^*p* = 0.066 comparing maximal differences between the tacrolimus- *vs* the metformin-treated mice (Mann-Whitney U test).

**Table 2:** Ratio of circulating Th17 (CD4+IL17A+): Th2 (CD4+IL4+) cells at gd 4.5 among experimental groups.

	HFD-dNONcNZO	NFD-NONcNZO	Metformin (200 mg/dL)	Tacrolimus (0.1 mg/kg)
^#^Animals	17	17	17	17
Range	0.35–1.96	0.16–0.95	0.08–0.94	0.06–0.69
Median ± ME	0.74 ± 0.03**	0.37 ± 0.02	0.42 ± 0.02	0.19 ± 0.02
Mean ± SDM	0.84 ± 0.43	0.44 ± 0.26^†^	0.41 ± 0.21^†^	0.25 ± 0.19^†^

**p < 0.0001 comparing group medians at alpha 0.05 (One-way ANOVA followed by Kruskal-Wallis test).

^†^*p* = 0.538 comparing maximal differences in group medians between the tacrolimus- *vs* the metformin-treated mice at 95% confidence interval (Mann-Whitney U test).

#### Systemic cytokines profile in the HFD-dNONcNZO mice at peri-conception

**Figure 7:**
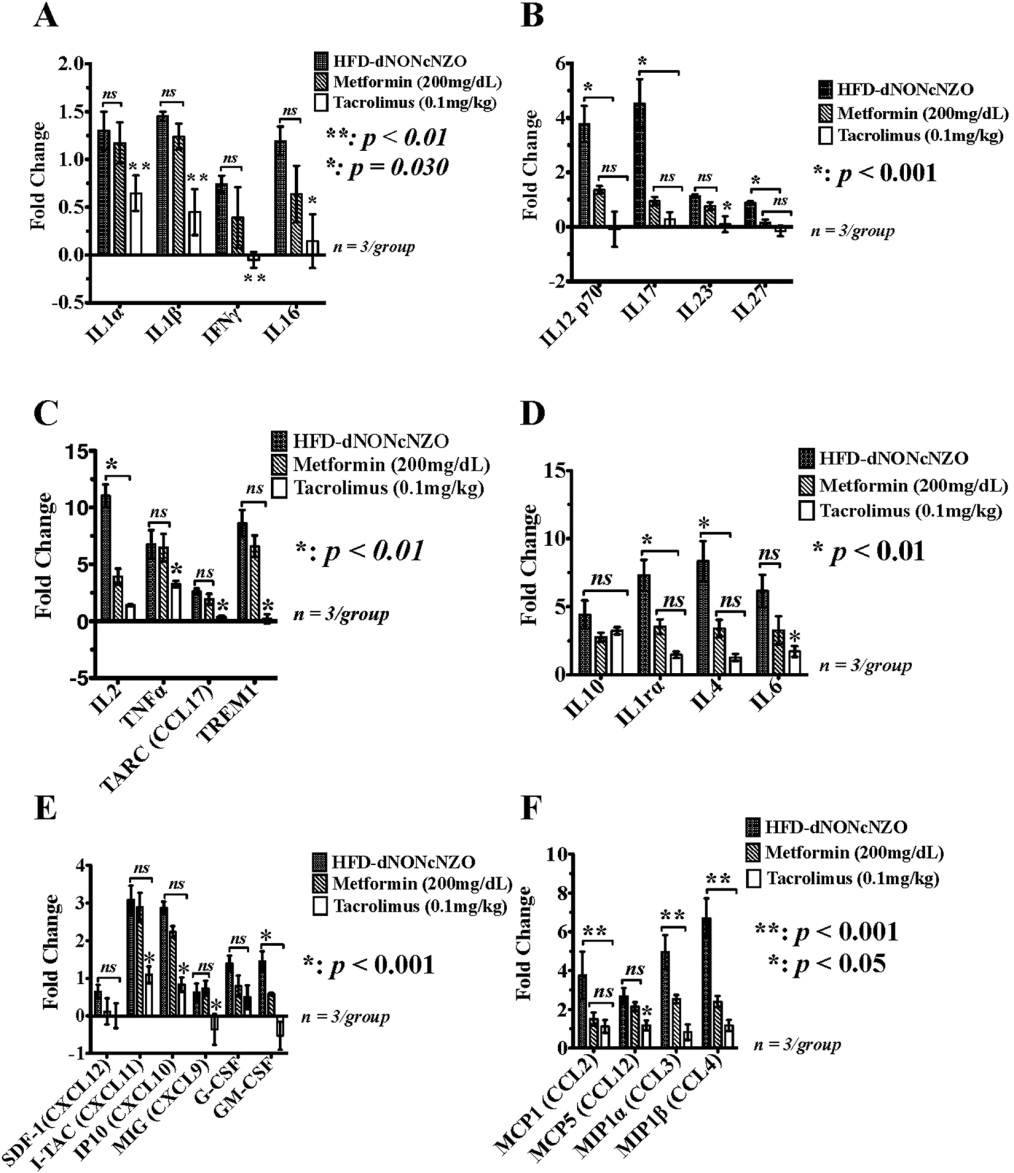
Effect of HFD and tacrolimus on circulating levels of pro-inflammatory and anti-inflammatory cytokines at gd 4.5 in the HFD-dNONcNZO mice. As opposed to the metformin-treated mice, the systemic use of tacrolimus monotherapy resulted in a wide-range suppression of cytokines and chemokines in the blood of treated HFD-dNONcNZO mice. Depicted in (**A**–**F**) are bar graph representations of mean ± SDM of the fold changes in cytokines suppression by use of tacrolimus or metformin. Effect of treatment was analyzed by one-way ANOVA followed by Scheffe’s *ad-hoc* test. A 1.5-fold change in the protein expression of the cytokine relative to that of the NFD-NONcNZO mice was considered significant (*p* < 0.01 at 95% confidence, n = 3/phenotype). Of the most significantly inhibited serum cytokines by the use of tacrolimus (0.1 mg/kg) were IL1α, IL1β and IFNγ (**A**), IL12p70, IL17, IL23 and IL27 (**B**), IL2, TNFα and its downstream chemokine ligands TARC and TREM1 (**C**), IL6, IL4 and IL1rα (**D**), GM-CSF and the CXC ligands 9,10 and 11 (**E**), as well as the monocyte-macrophage regulatory chemokines MIP-1α (CCL3), MIP-1ß (CCL4) and MCP5 (CCL12) (**F**), respectively.

Although PCOS is considered a state of a chronic systemic inflammatory condition^47^, nonetheless, many of the biomedical/clinical investigations into the etiology of PCOS have looked at ovarian follicular fluids for their contents of cytokines and chemokines, a few of these reports revealed an altered cytokine levels in the blood of women with PCOS^48^. This led many researchers to suggest that systemic immune dysregulation may be involved in the pathogenesis of PCOS^49^. As such, and given their prognostic values we analyzed the systemic expression of certain cytokines and chemokine ligands and their receptors in the HFD-dNONcNZO mice and those receiving tacrolimus or metformin by a murine specific immunoassay. As shown in Fig. 7, compared to the HFD-dNONcNZO values, our present data indicate significant inhibition in the systemic expression of IL16 (*p* < 0.05) and IFNγ (*p* < 0.01) (Fig. 7A), IL12p70, IL17, IL23 and IL27 (*p* < 0.001, Fig. 7B), IL2 and TNFα and its downstream chemokine ligands TARC (CCL17) and TREM1 (*p* < 0.01, Fig. 7C), as well as IL6 (*p* < 0.01, Fig. 7D), GM-CSF and the CXC ligands 9,10, and 11 (*p* < 0.001, Fig. 7E), the monocytes-macrophage regulatory chemokines MCP1 (CCL2), MIP-1α (CCL3) and MIP-1β (*p* < 0.001, Fig. 7F) and MCP5 (CCL12) (*p* < 0.05, Fig. 7F), respectively.

## Discussion

We have previously reported on the beneficial use of tacrolimus in mitigating severity and incidence of diabesity-associated maternal and fetal gestational adversities in the high-fat fed New Zealand Obese (NONcNZO) mice^28^. This mouse is a polygenic model of obesity-induced poor breeding performance with insulin resistance and hyperestrogenemia^36^. Among key contributing factors to subfertility in this mouse lineage are altered ovarian structure and function that are suggestive of PCOS^8^. Yet to be defined is whether the reported high rate of peri-implantation embryo loss in the HFD-dNONcNZO mouse^29^ is related to dysregulated ovarian and/or systemic immune responses to implantation. The obese and diabetic HFD-dNONcNZO mouse is known to express higher than control ratio of atretic ovarian follicles due to structural changes in the ovarian cortex preventing maturation and coordinated follicular development^8,50^. These changes include excessive initiation of follicular growth from the primordial follicle pool, followed by developmental failure and growth arrest at the medium-sized antral stage (5–10 mm)^51,52^. Although factors contributing to these ovarian changes in the HFD-dNONcNZO mice are yet to be identified, it has been suggested that similar changes are plausibly resulting from altered epigenetic regulation of the androgen receptor signaling due to aberrant ubiquitination during folliculogenesis as reported in the DHT-induced PCOS in rats^53^.

In the present study, important differences in localization of activated ovarian macrophages were found between treated and untreated HFD-dNONcNZO mice. Unlike the control NFD-NONcNZO and treated HFD-dNONcNZO mice and consistent with previous reports^54^, our histological and cellular studies of ovaries and follicular structures from untreated HFD-dNONcNZO mice showed extensive infiltration of the granulosa cell layer with CD11c + (M1) macrophages and depletion of the M2 ovarian macrophages at gd 4.5. This outcome is identical to the reported aberrancies in the ovarian M1:M2 macrophage ratios and altered tissue distribution of the M1 macrophages observed in the pre-ovulatory follicles in the DHT-induced PCOS in rats^13^. Although the exact mechanism(s) involved in the extensive infiltration of the antral and pre-ovulatory follicles with M1 activated ovarian macrophages in PCOS ovaries are yet to be fully comprehended, it is believed that aberrant expression of certain androgen-induced ovarian monocytes-derived cytokines, chemokines and adipokines are to be the culprit^13^. Lima *et al*.^13^ showed that independent of alterations in the systemic levels of the M1 activated macrophages, the monocytes-derived chemerin receptor (CMKLR1)-expressing M1 ovarian macrophages are predominantly involved in the processes of antral growth arrest and granulosa cell apoptosis reported in the DHT-induced PCOS in rats^13^. In our experience, analysis of ovarian cytokines and data obtained from the flow cytometry experiments showed a predominantly pro-inflammatory ovarian milieu characterized by aberrant expression of IL2 and IFNγ and its associated pro-inflammatory cytokines IL12p70, IL17, IL27 with elevated M1 ovarian macrophages (F4/80^+^ CD11c^+^) in the untreated HFD-dNONcNZO mice. IFNγ is the essential cytokine responsible for stimulating macrophage differentiation to the M1 phenotype^55,56^. Tacrolimus is known to inhibit IFNγ and IL2 in activated T cells^57^. Treatment with tacrolimus inhibited aberrant ovarian expression of the macrophage priming cytokines IL2 and IFNγ as well as IL12p70, IL7 and IL27 and the chemoattractant CXC ligands 9, 10 and 11 thereby inhibiting aberrant activation of M1 ovarian macrophages. Research on the role of macrophage polarization in obesity suggests that pathology results from imbalances between the pro-inflammatory and anti-inflammatory activated macrophage phenotypes^58^. Therefore, the mode of action of tacrolimus may involve controlling elevated levels of pro-inflammatory M1 macrophages to reduce inflammation and induce the alternative activation of ovarian macrophages. However, given that macrophages are critical for the induction of ovulation^1^, the perturbed macrophage localization pattern seen in the HFD-dNONcNZO mice is plausibly linked to poor ovulation and lack of coordinated folliculogenesis reported in this mouse model^8^. Therefore, besides suppressing the aberrantly inflamed ovaries, the beneficial effects of tacrolimus in the obese and diabetic HFD-dNONcNZO mice may be due to activation of homing receptors which recruit alternatively activated macrophages to developing follicles. This may partially explain the presently reported lack of suppressive effect of tacrolimus on the ovarian expression of the chemo-regulatory proteins MCP1 (CCL12), MCP5 (CCL12) and MIP-1β in the monocytes and macrophages found in tacrolimus-treated mice. On the other hand, it is generally accepted that metformin exerts beneficial effects by improving insulin sensitivity and decreasing inflammation, nevertheless our current data as well as others^59^ indicate that this sulfonylurea compound has differential effects on the immune and other organ systems in humans and mice. Treatment of obese and diabetic mice with metformin did not induce alternative activation of ovarian macrophages neither did it suppress aberrant ovarian production of the pleiotropic and pro-inflammatory molecules including TNFα, IL16 and the CXC ligands 9, 10 and 11. This further supports new and emerging evidence on the differential local and systemic actions of metformin as an immuno-regulatory compound capable of inducing paradoxical tissue-specific inflammatory responses in the obese subjects^59^.

Analysis of the percentages of circulating Th1 (CD4^+^IFNγ^+^), Th2 (CD4^+^IL4^+^) and the CD4^+^CD25^+^CD127^low^ regulatory T cells as well as the Th17 (CD4^+^IL17A^+^) cells revealed intriguing data suggesting the suitability of the HFD-dNONcNZO mice for studying obesity-induced immunological alterations and dysfunctional ovarian responses during early gestation in individuals with features of PCOS. This study revealed that obese and diabetic HFD-dNONcNZO mice are characterized by the peri-conceptional systemic abundance of Th1 (CD4^+^IFNγ^+^), Th2 (CD4^+^IL4^+^) and the Th17 (CD4^+^IL17A^+^) T cells which is associated with failure of expansion of circulating CD4^+^CD25^+^CD127^low^ regulatory T cells. Four consecutive weeks of mono-therapeutic interventions with tacrolimus (0.1 mg/kg) were successful in mitigating the impact of diabesity on the CD4^+^CD25^+^CD127^low^, CD4^+^ IFNγ^+^ and the CD4^+^IL17A^+^ T cells in the treated mice. Although CD4^+^IL17A^+^ T cells are notorious for graft rejection and mediating auto-immunity in type 1 diabetes mellitus^60^, nevertheless, both the CD4^+^CD25^+^CD127^low^ T and CD4^+^IL17A^+^ cells may participate in a suppressive activity and, as such, both are required for the induction and maintenance of tolerance during early pregnancy^22,61–63^. However, the translational value of our present findings on the positive effect of tacrolimus (0.1 mg/kg) or metformin (200 mg/dL) on the peri-conceptional expansions of CD4^+^CD25^+^ CD127^low^ T cells may be hard to reconcile in view of the conflicting data presently available on this contentious subject. Peri-conceptional expansion of CD4^+^IL4^+^ T cells is a defined signature of successful implantation in infertile women receiving tacrolimus^46^. Low dose tacrolimus is also known to favor the induction of functional CD4^+^CD25^+^FoxP3^+^ regulatory T cells in recipients of solid-organ transplants^35,64^. On the contrary, in a study conducted by Zhang and associates^65^ using *ex-vivo* cultured purified murine CD4^+^CD25^+^ Tregs obtained from otherwise healthy B6 mice from which there would be no therapeutic gain expected, increasing tacrolimus concentration from 0.1 to 1 and 10 ng/ml resulted in a one-fold reduction in IL17 mRNA. This dose-dependent *ex-vivo* inhibitory effects of this relatively high-dose tacrolimus on the differentiation and proliferation of Th17 cells and the inhibited expression of IL17 mRNA are believed to be consequential to Calcineurin-mediated suppression of T-cell receptor activation by tacrolimus^65^. Nevertheless, Lemster *et al*.^66^ reported that treatment with sub-clinical dose of tacrolimus has no significant effect on the proportion of circulating CD4^+^CD25^+^ T cells in patients in whom the mean trough plasma concentration of tacrolimus (FK506) ranged from 0.3 ± 0.2 to 0.5 ± 0.4 ng/ml.

In a recent study on polycystic ovary and circulating inflammatory markers, Zangeneh and associates^67^ reported significantly elevated systemic levels of IL1α and IL1β but astonishing low levels of IL17 in the serum of women with PCOS. Conversely, in a case control study on the clinical significance of ADAMTS proteinases, IL17, IL23 and IL33 in PCOS, Karakose and coworkers^37^ reported significantly higher levels of these cytokines in the serum of overweight (BMI ≥ 25) PCOS patients compared to controls and suggested a pathogenic role of these molecules in the etiology of PCOS. Similarly, Ozcaka *et al*.^68^ reported elevated IL17A among PCOS^68^. However, in a study conducted by Knebel and associates^69^ IL17A was found to be similar in PCOS patients and controls. IL17A is the prototypic product of Th17 T cells and is a potent effector regulating the expression and expansion of CD4^+^CD25^+^ T cells^70,71^. Nonetheless, Th17 T cells are a distinct subset of CD4^+^ regulatory T-cells essential for dominant immunologic rejection and play an important role in the acquisition of a transient state of maternal tolerance specific for parental alloantigens during early pregnancy^63,71^. Growth, differentiation and functions of Th17 T-cells are greatly influenced by T-cell Receptor (TCR) signaling^70^. Therefore, it is plausible that the peri-conceptional use of tacrolimus or metformin resulted in a transient state of TCR suppression that allowed for the expansion of the CD4^+^CD25^+^CD127^low^ Tregs while exerting suppressive effects on circulating CD4^+^IL17A^+^ cells in treated HFD-dNONcNZO mice. While this awaits further investigations, the presently reported paradoxical effect of diabesity on these two subsets of CD4^+^ T cells at peri-conception in the HFD-dNONcNZO mice is in conflict with previous studies showing increased prevalence and strong association between CD4^+^CD25^+^ Tregs and CD4^+^IL17A^+^ T cells and the expression of Th17 cytokines IL17, IL23 and the retinoid orphan nuclear receptor (RORC) in the blood and deciduae of women with recurrent spontaneous abortions^63^. This contradiction may, in part, be due to differences in timing of gestational sampling. Early phase of normal human pregnancy included that of the implantation phase is a transient inflammatory condition followed by a Th2/Th1 flip, in part in response to the expansion of CCR7^+^ CCR5^+^ paternal- and self- antigen-specific Tregs for the induction of tolerance^72^.

The systemic cytokine milieu in the untreated HFD-dNONcNZO mice was predominantly cytotoxic and pro-inflammatory in nature with the presence of an aberrantly expressed IL6, IL16, IL12p70, IL17A, IL23 and IL27, TNFα and TARC (CCL17) at peri-conception. This has important implications in translating the presently reported data on the alterations in the peri-conceptional percentages of Th1, Th2, Th17 and the CD4 + CD25 + CD127low Tregs in the untreated and treated HFD-NONcNZO mice. It has been hypothesized that conditions typically favor the development of CD4 + CD25 + Tregs and promote immunological balance can be subverted by inflammatory signals that support the generation of Th17 cells^71^. This has been demonstrated in *ex vivo* cultured murine CD4+ T cells treated with cytokines IL6, IL12 and IL23 with or without the presence of TGFβ^71^. IL6 is known to divert naïve CD4+ T cells from a regulatory to an inflammatory pathway whereas TGFβ induces the expression of Th17 cells^73^. In inducing its pan-suppressive effect, tacrolimus inhibits calcineurin in Th17 cells, blocks dephosphorylation and nuclear translocation of NFAT cytoplasmic-1 protein (NFATc), inhibits transcriptional activation of the IL-17α gene and reduces the expression of IL-17A allowing the suppression of allograft rejection^74^. This wide-range of the tacrolimus-induced immunosuppression was associated with improved implantation and pregnancy rates among infertile women with elevated Th1:Th2 ratios^46^ as well as among the obese and diabetic HFD-dNONcNZO mice^29^. On the other hand, consistent with the human studies on the anti-inflammatory effect of metformin in women with PCOS^75^, the use of this anti-diabetic agent inhibited systemic IL17A production in activated T cells, suppressed aberrant Th1:Th2 and Th17: Th2 cell ratios and resulted in a peri-conceptional expansion of the CD4 + CD25 + CD127low Tregs in treated HFD-dNONcNZO mice. However, unlike the *ex-vivo* effect of metformin in inducing enzymatic phenotypic shift toward the alternative activation of macrophages reported in human studies^76^, the currently prescribed dosages of metformin at 200 mg/dL/day did not induce the expression of the M2 (F4/80 + CD206+) ovarian macrophages in the HFD-dNONcNZO mice. This is intriguing since it resembles the effect of metformin (60 μM) on macrophage polarization in tumour tissues^77^. Evidence from *ex-vivo* studies on murine bone marrow-derived macrophages and human monocytes-derived macrophages revealed that metformin modulates the expression of inflammatory cytokines through the activation of the AMPK and suppression of NFκB pathway promoting macrophage polarization to an anti-inflammatory phenotype^76,78^. It is generally held that the M2 phenotype to be induced by cytokines including IL-4, IL-10 and IL-13, and the M1 phenotype be induced by cytokines including IFN-γ^79^. Although treatment with metformin is anticipated to activate AMPK pathway and subsequently restricts IFN-γ signaling^80^, our current therapeutic intervention with metformin (200 mg/dL/day) did not result in a significant inhibition to ovarian and systemic secretion of this pleotropic cytokine. This may be due to the dose or duration of the metformin treatment prescribed in the present study. Further studies exploring the effect of higher dosage and longer duration of monotherapeutic intervention with metformin on ovarian macrophage activation may be warranted.

Collectively, it is tempting to speculate that the observed positive effect of the pre-pregnancy use of tacrolimus on CD4^+^CD25^+^CD127^low^ Tregs and CD4^+^IL17A^+^ T cells and the subsequent normalization of the Th1:Th2 and Th17:Th2 cell ratios may plausibly be secondary to the induction of a favorable systemic milieu generated by the normal progression of early gestation in treated subjects. It is also imperative to suggest that the wide range of actions of tacrolimus on the CD4^+^CD25^+^ CD127^low^ Tregs and the CD4^+^IL17A^+^ T cells are largely circumstantial in nature requiring additional priming signals likely generated through the pan-cytokine suppressive effects of this macrolide immunosuppressant. Further studies may be warranted to elucidate on the exact mode of action of tacrolimus in promoting the expansion of the CD4^+^CD25^+^ CD127^low^ T cells at peri-conception in individuals with PCOS. Of importance is to consider evaluating the effects of tacrolimus on local ovarian and systemic levels and signaling pathways of TGFβ and its effects on the functional proliferation and differentiation of the CD4^+^CD25^+^ CD127^low^ T cells in PCOS.

In conclusion, this study has presented yet another evidence for the beneficial use of tacrolimus in the restoration of functional ovarian and systemic immune milieux conducive to early gestational success in individuals with PCOS. Our work supports the idea that obese and diabetic female subjects with PCOS have an unbalanced Treg/Th17 cell ratio which may contribute to early gestational complications partly through the acceleration of dysfunctional ovarian folliculogenesis leading to premature ovarian failure and early pregnancy termination. Future research may identify the homing factors and signaling pathways involved in the systemic cross-talks governing the restricted peri-conceptional expansion of CD4^+^CD25^+^ CD127^low^ Tregs and the aberrant expression of CD4^+^IL17A^+^ T cells in recruiting activated ovarian macrophages to various locations within the polycystic ovary. Elucidation of therapeutic strategies for the selective pre-pregnancy inhibition of IL17A in promoting peri-conceptional expansion of Tregs and/or attenuating Th17 subsets may have important implications for therapy of PCOS-associated female infertility.

## Supplementary information

Supplementary Dataset 1

## Data Availability

All experimental data pertaining to this manuscript can be made available to the scientific community upon request to the corresponding author.
