# Supplementary material for: Low-Dose Tacrolimus Prevents Dysregulated Peri-Conceptional Ovarian and Systemic Immune Cellular Homeostasis in Subjects with PCOS
Source: Sci Rep. 2019 Apr 25;9:6528. doi: 10.1038/s41598-019-42960-x (PMC6484102; doi:10.1038/s41598-019-42960-x)
Supplement: Supplementary file 1 — Supplementary Dataset 1 [file 41598_2019_42960_MOESM1_ESM.docx]

**Supplemental Materials and Methods, Tables and Figures for:**

**Low-Dose Tacrolimus Prevents Dysregulated Peri-Conceptional Ovarian and Systemic Immune Cellular Homeostasis in Subjects with PCOS**

By

Ahmad J.H. Albaghdadi^a^, Carolyn Ann Feeley^a^ and Frederick W.K. Kan^a,1^

^a^ Department of Biomedical and Molecular Sciences, Faculty of Health Sciences, Queen’s University, Kingston, Ontario, Canada K7L 3N6

^1^Corresponding author: Dr. Frederick W.K. Kan

Address: Department of Biomedical and Molecular Sciences, Faculty of Health Sciences, Queen’s University, Kingston, Ontario, Canada, K7L3N6

Email: kanfwk@queensu.ca

Telephone: +1 613 533-2863

***Supplemental Materials and Methods***

***Gating strategies for peripheral blood lymphocytes and ovarian macrophages:***

***1.*** ***Gating for lymphocytes****:*

A maximum of 1x 10^6^ fixed and permeabilized lymphocytes were stained with the live/dead discriminator stain 7 Aminoactinomycin D (7-AAD) and sorted based on their staining and scatter characteristics (i.e. forward (FSC) *vs* side scattering (SSC)) (Supplemental Figures S2-A and B). Positive signal for each staining was established using appropriate isotype controls and unstained. SSC/pulse-width was applied to exclude potential doublets and events that could represent more than 1 cell. Proportions and absolute numbers of the CD4^+^ and CD8α^+^ T cells were calculated after primary gating was performed on the CD4^+^ *v*s SSC, CD8α^+^ *vs* SSC and CD4 *vs* CD8α^+^ (Supplemental Figure S2-C). Subsequent gating was performed on the CD4^+^ *vs* CD25^+^, CD4^+^ *vs* CD127^+^ and CD25^+^ *vs* CD127^+^ (Supplemental Figures S2-D) allowing for the clear discrimination of CD4^+^CD25^+^CD127^low^ (Supplemental Figures S2-D). The expression of IFNγ, IL4 and IL17A on the CD4 positive gate was quantified by determining mean fluorescence intensity (MFI). The MFI was quantified as a ratio of mean fluorescence intensity for IFNγ, IL4 or IL-17A to MFI for appropriate isotype control. In gating for isotype controls, histograms were generated by cell count *vs* CD4^+^, cell count *vs* CD25^+^, cell count *vs* CD127^low^, cell count *vs* IFNγ, cell count *vs* IL4 and cell count *vs* IL17A, respectively (Supplemental Figures S2-E). The results are expressed as mean ± SDM of the Treg cells in total lymphocyte population.

***2.*** ***Gating for ovarian macrophages****:*

As shown in Supplemental Figure S3, for the detection of the activation status and subsets of ovarian macrophages, populations of fixed and permeabilized cells were first selected in light scatter (FSC *vs* SSC: **A**) and sorted for their staining for the live/dead discriminator 7-AAD (**B**). SSC/pulse-width was applied to exclude potential doublets (**C**). Primary gating was then performed on the CD45^+^ *vs* FSC (**D**) and subsequent gating was performed on the populations CD45^+^ *vs* F4/80^+^ (**E**) F4/80^+^ *vs* CD206^+^ (**F**), F4/80^+^ *vs* CD11c^+^ (**G**) and CD11C^+^ *vs* CD206^+^ (H). This allowed the clear separation of activated ovarian F4/80^+^ CD206^+^ CD11c^+^ resident cells. For gating of isotype controls, histograms were generated by cell count *vs* all the lymphocytes and/or macrophage activation markers used in this study. Subsets of activated ovarian macrophages are expressed as mean ± SDM in total ovarian F4/80^+^ population (Supplemental Table S4).

**Supplemental Table S1: Mouse models, therapeutic interventions, dietary and husbandry conditions reported in this study.**

**Supplemental Table S2: Fluorophores conjugated primary antibodies and controls used in this study.**

**Supplemental Table S3. Effect of the HFD, tacrolimus and metformin on the circumference (mm) of antral ovarian follicles at gd 4.5 among experimental groups**

**Supplemental Table S4: Time-course alterations to percentages of M1 (F4/80^+^ CD206^+^ CD11c^+^) *vs* M2 (F4/80^+^ CD206^+^ CD11c^-^) polarized ovarian macrophages at gd 2.5, 4.5 and 6.5, respectively, in the NONcNZO mice.**

**Supplemental Table S5: Percentages of circulating CD25^+^CD127^-low^ (%CD4^+^) cells at gd 4.5 among experimental groups.**

**Supplemental Table S6: Percentages of circulating Th1 (CD4^+^IFNγ^+^) cells at gd 4.5 among experimental groups.**

**Supplemental Table S7: Percentages of circulating Th2 (CD4^+^IL4^+^) cells at gd 4.5 among experimental groups.**

**Supplemental Table S8: Percentages of circulating Th17 (CD4^+^IL17A^+^) cells at gd 4.5 among experimental groups**

**Supplemental Figure S1: Weight gain over time and tacrolimus and metformin schedules.**

The NONcNZO/LtJ mice were weaned onto 60% kcal% high-fat diet (HFD) for 17 consecutive weeks and received tacrolimus (0.1mg/kg) subcutaneously for four successive weeks between HFD weeks 11- 14 (corresponding to weeks 15-18 of age). Metformin (200mg/dL *ad libitum*) was administered daily from age weeks 15 to 20 to the metformin-treated control HFD-NONcNZO mice. A group of the NONcNZO/LtJ mice (n= 17) were weaned onto 6% fat diet high in proteins (20% proteins w/w) and were used as normative (normoglycemic) controls (also referred to as NFD-NONcNZO). There were no signs of the tacrolimus toxicity such as significant weight loss, tremors or diarrhea. All HFD mice with body mass ≥ 25gm and impaired glucose tolerance at the end of week 3 HFD were considered obese and diabetic HFD-dNONcNZO. All NFD-NONcNZO mice did not become obese or diabetic at experimental end-points while on protein-rich 6% fat diet. *ns=* statistically not significant (*p* > 0.05 at 95% confidence).

**Supplemental Figure S2: Gating tree for isolated peripheral blood lymphocytes.**

Isolated fixed and permeabilized lymphocytes were stained with the live/dead discriminator stain 7 Aminoactinomycin D (7-AAD) and sorted based on their staining and scatter characteristics (A and **B**). SSC/CD4^+^ and SSC/CD8α^++^gates with a maximum of 1x 10^6^ cells were created and allowed the clear distinction of the CD4^+^ CD8α^+^ lymphocytes in biexponential logarithmic transformations after background fluorescence subtraction and error compensation using unstained, stained and IgG2a, k isotype controls, respectively (**C**). Biexponential CD4^+^ *vs* CD25^+^ and CD4^+^ *vs* CD127^+^ identifying selective subsets of CD4^+^ CD25^+^, CD4^+^ CD127^+^ and CD25^+^ CD127^low^ T cells (**D**). The immunophenotypic identification of the Th1 (CD4^+^ IFNγ^+^), Th2 (CD4^+^ IL4^+^) and Th17 (CD4^+^ IL17A^+^) T cells was independently performed on a CD4^+^ gate after staining with a mouse Th1/Th2/Th17 Phenotyping Kit including CD4 PerCP-Cy5.5, IFNγ FITC, IL-4 APC and IL-17A PE (**E**).

**Supplemental Figure S3: Gating for isolated ovarian macrophages.**

Gating on live cells was determined by light scatter characteristics (**A**) and the viability dye 7-AAD was used as the lived/dead discriminator (**B**). SCC *vs* pulse-width was applied to exclude events that could present more than one time (**C**). Primary gating on the CD45^+^ *vs* SCC followed with a maximum of 10,000 cells events was created (**D**). Subsequent gating for CD45^+^ *vs* F4/80^+^ (E), F4/80^+^ *vs* CD206^+^ (**F**), F4/80^+^ *vs* CD11c+ (**G**) and CD11c^+^ *vs* CD206^+^ (**H**) followed. In gating for isotype controls, histograms were generated by cell count *vs* all the macrophage activation markers used in this study. Subsets of activated ovarian macrophages are expressed as mean ± SDM in total ovarian F4/80^+^ population.

**Supplemental Figure S4: Effect of tacrolimus treatment on activated ovarian macrophages**.

**A**- **D** are representative flow-cytometric histograms depicting mean fluorescence intensity (MFI) and the median percentage (%) of F4/80+ ovarian macrophages from an untreated HFD-dNONcNZO mouse (**A**), an NFD-NONcNZO control mouse (**B**), a metformin-treated HFD-dNONcNZO mouse (**C**) and a tacrolimus-treated HFD-dNONcNZO (**D**) at postcoital/gestational day (gd) 4.5. CD45+ cells were first gated for their expression of the macrophage surface marker F4/80 and were subsequently analyzed for their expression of the activation markers CD11c and CD206. Untreated HFD-dNONcNZO ovaries expressed higher percentages of F4/80 positive (**A**) and M1 (CD11c+ CD206+) activated macrophages (% expressed in parenthesis in quadrant **B2** in **Ai**). Conversely, percentages of alternatively activated ovarian F4/80 + (**B**- **C**) and M2 (CD11c- CD206+) activated cell population of ovarian macrophages {(%) in quadrants **B4** in **Bi**- **Ci**} were predominantly seen at gd 4.5 in the NFD-NONcNZO and the tacrolimus-treated mice. It is yet to be determined why the use of metformin (200mg/dL) did not induce a significant expansion of the ovarian M2 population in treated HFD-dNONcNZO mice (% of CD11c- CD206+ M2 population in quadrant **B4** in **Di**). Isotropic control staining is shown in red in **A- D**, respectively.

**Supplemental Figure S5: Effect of tacrolimus, metformin and HFD on circulating CD25^+^ CD127^low^ lymphocytes in the HFD-dNONcNZO mice.**

**A**: representative flow-cytometric dotplots of the CD25^+^ CD127^low^ lymphocytes obtained from untreated HFD-dNONcNZO mice, their normative control and those receiving metformin or tacrolimus. Lymphocytes were gated for their dual expression of their cell-surface receptors CD25 (IL2rα) and CD127 (IL7rα) and percentages (%) given in parentheses of CD25^+^ CD127^low^ T cells were calculated based on the proportion of the respective population that is positive and/or negative for CD25 and CD127 toward the total CD4^+^ lymphocytes. The mean fluorescent intensity (MFI) represented in shaded vertical columns in the flow-cytometric histograms in **B** was calculated using unstained, isotype controls and stained, respectively.

Cell sorting and immunophenotyping were performed on a Beckman Coulter FC500 flow cytometer using Summit software 4.3 and post-acquisition analysis was performed using FlowJo™ V 10.4.2. FACS experiment for the antibodies as well as isotype controls for each subject was independently performed once.

**Supplemental Figure S6: Peri-conceptional expression of IFNγ, IL4 and IL17A in the CD4+ T cells in the HFD-dNONcNZO mice**.

Flow-cytometric scatterplot representing percentages (%) of IFNγ expressing CD4+ cells (**A**), (%) of IL4 expressing CD4+ cells (**B)** and **(**%) of IL17A expressing CD4+ (**C**) in lymphocytes in the untreated HFD-dNONcNZO mice (n= 17) *vs* control NFD-NONcNZO mice (n = 17) and those receiving metformin or tacrolimus (n= 17/group). Each dotplot represents 10 000 lymphocyte events gated for CD4+ expression and cell % given in parentheses were calculated based on the proportion of the respective population that is positive for IFNγ, IL4 and IL17A toward the total lymphocytes. Compared to control, untreated HFD-dNONcNZO mice have significantly higher % of CD4^+^IFNγ^+^ T cells (mean difference = 9.95, *p* = 0.00002, t = 5.491; 95% confidence interval = 4.749 – 15.161). Treatment with tacrolimus rather than metformin significantly inhibited an aberrant expression of the IFNγ secreting CD4^+^ T cells in the HFD-dNONcNZO mice cells (mean difference = 14.253, *p* < 0.00001, t = 7.861; 95% confidence interval = 9.047 – 19.458) (n= 17/group). No statistically significant differences were observed between tacrolimus and metformin in their effects on the CD4^+^IL4^+^ and the CD4^+^IL17A^+^ cells.

Histograms representing the mean fluorescence intensity (MFI) of IFNγ, IL4 and/or IL17A in CD4^+^ T cells were generated by cell count *vs* IFNγ, cell count *vs* IL4 and cell count *vs* IL17A, respectively (**D**)

**Supplemental Figure S7: Cytokine Array Proteome Profiler^TM^ Array in the HFD-dNONcNZO mice**.

Representative images of the Cytokine Array Proteome Profiler^TM^ Array (Mouse Cytokine Array Panel A Array Kit, Catalog number ARY006, R&D Systems) depicting peri-conceptional alterations to 26 ovarian and serum cytokines and chemokines in the HFD-NONcNZO mice examined in the present investigation as described in Figures 4 and 7. Starting at 1-2 and ending at 23-24, counting for each of the duplicate dot-blots starts from left to right in the direction of the arrow. Data in bargraphs in Figures 4 and 7 were the outcome of averaging pixel intensities of three blots obtained per experimental phenotype. Blots from all experimental groups were exposed on one film for 1 and 3 minutes, respectively according to the manufacturer’s instructions and were made explicit by white spaces as shown. The method used in cytokines/chemokines identification and representative one-minute exposures are shown in Related Supplemental Figures S1-S3, respectively.

**Supplemental Table S1: Mouse models, therapeutic interventions, dietary and husbandry conditions reported in this study.**

| Mouse Model | Source | Diet | Therapeutic Intervention | Numbers |
| --- | --- | --- | --- | --- |
| NONcNZO10/LtJ | The Jackson Lab. (stock # 004456M) | 60% kCal high fat diet (D12492, Research Diets Inc., NJ, USA) | Vehicle **^a^**  (Castor oil: ethanol mix) | 17 |
| NONcNZO10/LtJ |  |  | Tacrolimus  (0.1mg/kg s.c. q2d) * | 17 |
| NONcNZO10/LtJ |  |  | Metformin (200mg/dL) **^b^** | 17 |
| NONcNZO10/LtJ | The Jackson Lab. (stock # 004456M) | 20% fortified protein pellet diet (5K52 LabDiet^®^) | Vehicle **^c^** | 17 |
| NONcNZO10/LtJ | The Jackson Lab. (stock # 004456M) | 20% fortified protein pellet diet (5K52 LabDiet^®^) | None **^d^** | 10 |

*: Administered on an alternate day regimen from week 11- 14 HFD corresponding to age weeks 15-18, respectively, prior to mating.

**^a^**: Received vehicle for tacrolimus and referred to as HFD-dNONcNZO.

**^b^**: Received metformin (200mg/dL *ad libitum*) from age weeks 15 to 20.

**^c^**: Used as normative (normoglycemic) controls (referred to as NFD-NONcNZO).

**^d^**: Untreated non-pregnant normoglycemic reference cohort for assessing effect of vehicle on body mass in the NFD-NONcNZO (referred to as NFD-NONcNZO null).

- Mice were housed in a decontaminated barrier facility under standard sanitation, disinfection and sterilization practices as approved by Queen’s University Animal Care Services.
- The animal room environment and photoperiod were as follows: temperature 20 ± 3**°**C; humidity 30% to 70%; 12hour light/dark photoperiod (lights on at 07:00AM).

**Supplemental Table S2: Fluorophores conjugated primary antibodies and controls (Bio-Markers) used in this study.**

| Bio-Marker | Fluorophores | Provider/ Batch Number | Specificity |
| --- | --- | --- | --- |
| Anti-CD45 | PE_Texas Red  (PE/TR) | ThermoFisher Scientific (MHCD4517) | Human, Mouse |
| Anti- F4/80 | PE | Biolegend (123109) | Mouse, Rat and Human |
| Anti-CD206 | APC | BioLegend (141707) | Mouse |
| Anti-CD11c | PE/Cy7 | BioLegend (117317) | Mouse |
| IgG2a κ isotype control | PE/Texas Red (PE/TR) | ThermoFisher Scientific (R2a17) | Rat, Mouse |
| IgG2a κ isotype control | PE | BioLegend (400507) | Rat, Mouse |
| IgG2a κ isotype control | APC | BioLegend (400511) | Rat, Mouse |
| IgG1 λ isotype control | PE/Cy7 | BioLegend (401908) | Rat, Mouse |
| Anti-CD25 | PE/Cy7 | BD Biosciences (552880) | Mouse, Rat |
| Anti-CD4 | APC | BioLegend (100411) | Mouse |
| Anti-CD127 | PE | BD Biosciences (557938) | Mouse, Human |
| Anti-CD8α | Brilliant Violet 785™ | BioLegend (100749) | Mouse, Rat |
| IgG2a κ isotype control | Brilliant Violet 785™ | BioLegend (400545) | Mouse, Rat |
| IgG2a κ isotype control | Alexa Fluor 488 | BioLegend (400625) | Rat, Mouse |

**Supplemental Table S3. Effect of the HFD, tacrolimus and metformin on the diameter (mm) of antral ovarian follicles at gd 4.5 among experimental groups**

|  | HFD-dNONcNZO | NFD-NONcNZO | Metformin (200mg/dL) | Tacrolimus 0.1mg/kg |
| --- | --- | --- | --- | --- |
| # Animals (#Ovarian Sections Examined) | **8 (24)** | **6 (28)** | **8 (34)** | **8 (36)** |
| Range | **0.48 – 1.23** | **1.15 – 1.72** | **0.82 – 1.93** | **1.15 – 3.39** |
| Median ± ME | **0.66 ± 0.01^a^** | **1.25 ± 0.03** | **1.24 ± 0.03** | **1.58 ± 0.07** |
| Mean ± SDM | **0.59 ± 0.05** | **1.19 ± 0.09** | **1.13 ± 0.09 †** | **2.07 ± 0.21** |

| **^a^***: P* < 0.0001 independently comparing group medians at alpha 0.05 (Kruskal-Wallis ANOVA test).  **†**: *P* = 0.0045 comparing maximal differences between the tacrolimus- *vs* the metformin-treated mice (Mann-Whitney U test). |
| --- |

**Supplemental Table S4: Time-course alterations to percentages of M1 (F4/80^+^ CD206^+^ CD11c^+^) *vs* M2 (F4/80^+^ CD206^+^ CD11c^-^) polarized ovarian macrophages at gd 2.5, 4.5 and 6.5, respectively, in the NONcNZO mice. Data are presented as mean ± SDM.**

|  | M2: CD206+ CD11c- (% of total F4/80+) | | | M1: CD206+ CD11c+ (%of total F4/80+) | | |
| --- | --- | --- | --- | --- | --- | --- |
| **gd** | **2.5** | **4.5** | **6.5** | **2.5** | **4.5** | **6.5** |
| **HFD-dNONcNZO** | **1.62 ± 0.17 ^a^** | **0.69 ± 0.18 ^a, b^** | **1.01 ± 0.05^a^** | **7.87 ± 0.12**** | **7.95 ± 0.14**** | **9.75 ± 0.39**** |
| **NFD-NONcNZO** | **5.78 ± 0.17** | **9.81 ± 0.38** | **6.85 ± 0.15** | **2.34 ± 0.41** | **3.38 ± 0.15** | **5.12 ± 0.22** |
| **Metformin**  **(200mg/dL)** | **1.05 ± 0.21†** | **1.11 ± 0.56^b^, ††** | **2.48 ± 0.33 ††** | **3.93 ± 0.22** | **3.81 ± 0.46** | **5.12 ± 0.22** |
| **Tacrolimus**  **(0.1mg/kg)** | **2.29 ± 0.19^d^** | **8.69 ± 0.19** | **9.05 ± 0.50** | **0.66 ± 0.07^c^** | **2.26 ± 0.59*** | **1.72 ± 0.18^c^** |

**: *P* < 0.001 comparing the variance of the HFD-dNONcNZO values with the treated and their normative control.

*: *P* < 0.05 at alpha 0.05 comparing median and average values of the tacrolimus-treated to that of the metformin-treated and their normative control by Kruskal-Wallis test

**^a^**: *P* < 0.001 comparing source of variations between and within groups at 95% confidence using one-way ANOVA flowed by Neuman-Keuls and Fisher LSD.

**^b^**: *P =* 0.063 at alpha 0.05 comparing group medians and average values of the HFD-dNONcNZO mice and those receiving (Kruskal-Wallis test)

**^c^**: *P* < 0.05 at alpha 0.05 comparing median and average values of the tacrolimus-treated to that of the metformin-treated (Kruskal-Wallis test)

**^d^**: *P* = 0.003 compared to the NFD-NONcNZO control values at 95% confidence (Neuman-Keuls test)

†: *P* = 0.042 compared to the tacrolimus-treated at 95% confidence (Neuman-Keuls test)

††: *P* < 0.001 compared to the tacrolimus-treated at 95% confidence (Neuman-Keuls test)

**Supplemental Table S5. Percentages of circulating CD25^+^CD127^low^cells (%CD4^+^) at gd 4.5 among experimental groups**

|  | HFD-dNONcNZO | NFD-NONcNZO | Metformin (200mg/dL) | Tacrolimus 0.1mg/kg |
| --- | --- | --- | --- | --- |
| # Animals | **17** | **17** | **17** | **17** |
| Range | **6.10 – 21.75** | **16.01 – 39.75** | **20.63 – 36.45** | **12.09 – 31.30** |
| Median ± ME | **16.82 ± 0.34**** | **27.65 ± 0.46**** | **29.68 ± 0.33**** | **23.05 ± 0.41*** |
| Mean ± SDM | **15.97 ± 4.84** | **28.16 ± 6.62** | **29.01 ± 4.86 †** | **22.15 ± 5.87 †** |

| ***: p* < 0.0001 independently comparing group medians at alpha 0.05 (Kruskal-Wallis test).  **: p* = 0.0168 comparing maximal differences between the tacrolimus-treated *vs* untreated mice (Mann-Whitney U test).  †: *p* = 0.0061 comparing maximal differences between the tacrolimus- *vs* the metformin-treated mice (Mann-Whitney U test). |
| --- |

**Supplemental Table S6. Percentages of circulating Th1 (CD4^+^IFNγ^+^) cells at gd 4.5 among experimental groups**

|  | HFD-dNONcNZO | NFD-NONcNZO | Metformin (200mg/dL) | Tacrolimus 0.1mg/kg |
| --- | --- | --- | --- | --- |
| # Animals | **17** | **17** | **17** | **17** |
| Range | **19.50 – 39.60** | **10.9 – 27.95** | **12.25 – 33.80** | **7.15 – 19.61** |
| Median ± ME | **30.15 ± 0.46**** | **19.6 ± 0.38**** | **22.85 ± 0.39*** | **16.85 ± 0.31**** |
| Mean ± SDM | **29.11 ± 6.28** | **19.15 ± 5.19** | **24.04 ± 5.35 †** | **14.85 ± 4.08 †** |

| **: *P* < 0.0001 independently comparing group medians at alpha 0.05 (Kruskal-Wallis test).  *: *P* = 0.0219 comparing maximal differences between the metformin-treated *vs* the untreated HFD-dNONcNZO mice at 95% confidence (Mann-Whitney U test).  †: *P* < 0.001 comparing maximal differences between the tacrolimus- *vs* the metformin-treated mice at 95% confidence (Mann-Whitney U test). |
| --- |

**Supplemental Table S7. Percentages of circulating Th2 (CD4^+^IL4^+^) cells at gd 4.5 among experimental groups**

|  | HFD-dNONcNZO | NFD-NONcNZO | Metformin (200mg/dL) | Tacrolimus 0.1mg/kg |
| --- | --- | --- | --- | --- |
| # Animals | **17** | **17** | **17** | **17** |
| Range | **15.76 – 53.28** | **6.85- 31.60** | **11.93 – 40.83** | **6.05 – 27.63** |
| Median ± ME | **38.32 ± 0.68**** | **21.75 ± 0.53**** | **31.80 ± 0.65*** | **16.15 ± 0.49**** |
| Mean ± SDM | **38.11 ± 9.33** | **21.07 ± 7.17** | **27.89 ± 8.81†** | **16.81 ± 6.70†** |

| ***: P<* 0.0001independently comparing group medians at alpha 0.05 (Kruskal-Wallis test)  **: P=* 0.0061 comparing maximal differences between the metformin-treated *vs* untreated HFD-dNONcNZO mice at alpha 0.05 (Mann-Whitney U test).  †: *P* = 0.0024 comparing maximal differences between the tacrolimus- *vs* the metformin-treated mice at alpha 0.05 (Mann-Whitney U test) |
| --- |

**Supplemental Table S8. Percentages of circulating Th17 (CD4^+^IL17A^+^) cells at gd 4.5 among experimental groups**

|  | HFD-dNONcNZO | NFD-NONcNZO | Metformin (200mg/dL) | Tacrolimus 0.1mg/kg |
| --- | --- | --- | --- | --- |
| # Animals | **17** | **17** | **17** | **17** |
| Range | **35.11 – 71.70** | **9.41 – 42.18** | **13.04 – 45.33** | **5.19 – 24.38** |
| Median ± ME | **52.10 ± 0.85 **** | **24.44 ± 0.82*** | **28.52 ± 0.69** | **15.66 ± 0.44*** |
| Mean ± SDM | **52.52 ± 11.60** | **24.11 ± 11.07** | **28.02 ± 9.36†** | **14.53 ± 5.92†** |

| ***: P<* 0.0001independently comparing group medians at alpha 0.05 (Kruskal-Wallis test)  *: *P* = 0.0503 comparing control NFD-NONcNZO *vs* the tacrolimus- treated mice at alpha 0.05 (Kruskal-Wallis test)  †: *P* = 0.0021 comparing maximal differences between the tacrolimus- *vs* the metformin-treated mice at alpha 0.05 (Mann-Whitney U test) |
| --- |

**Supplemental Figure S1**

**
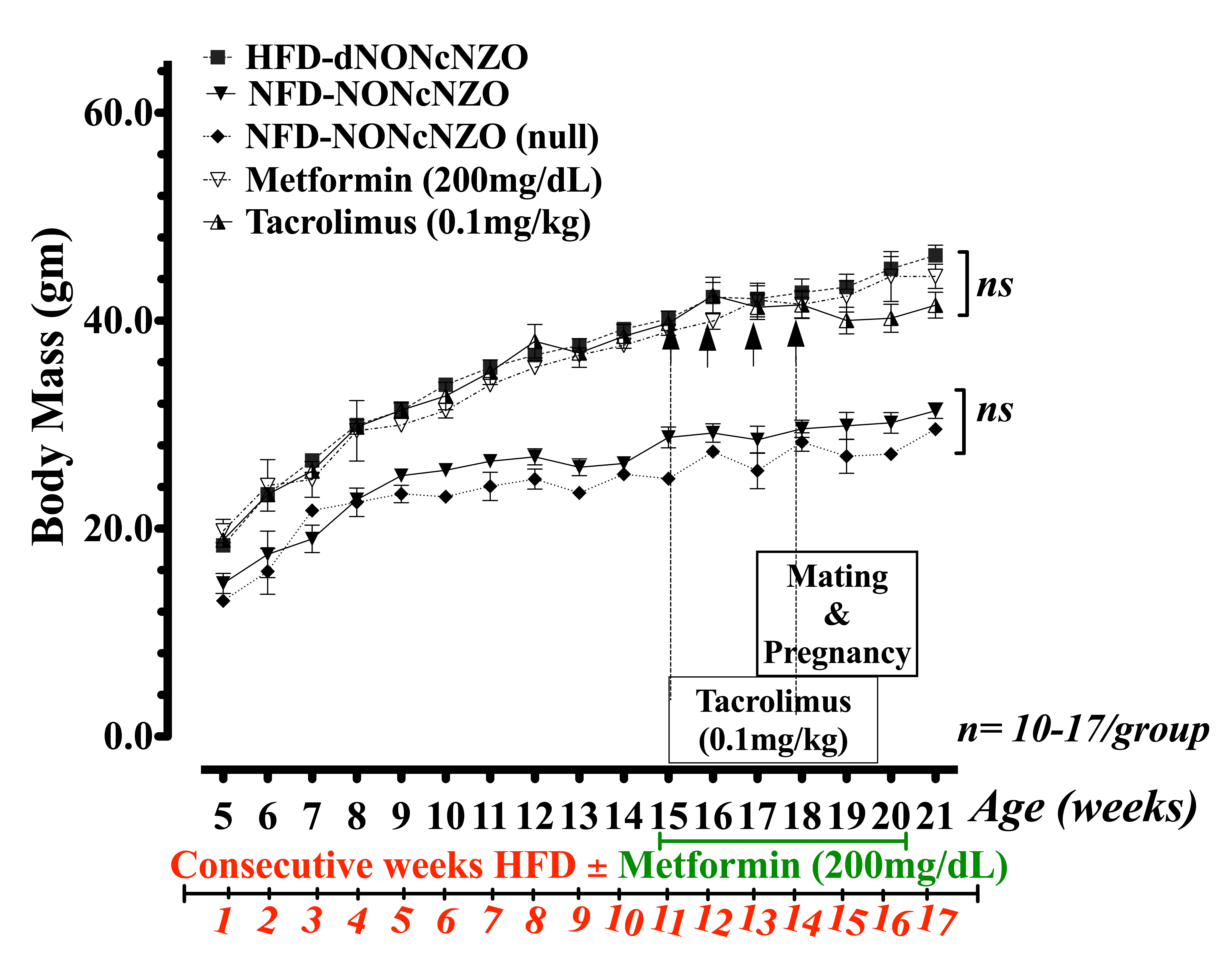
**

**Supplemental Figure S2**

**
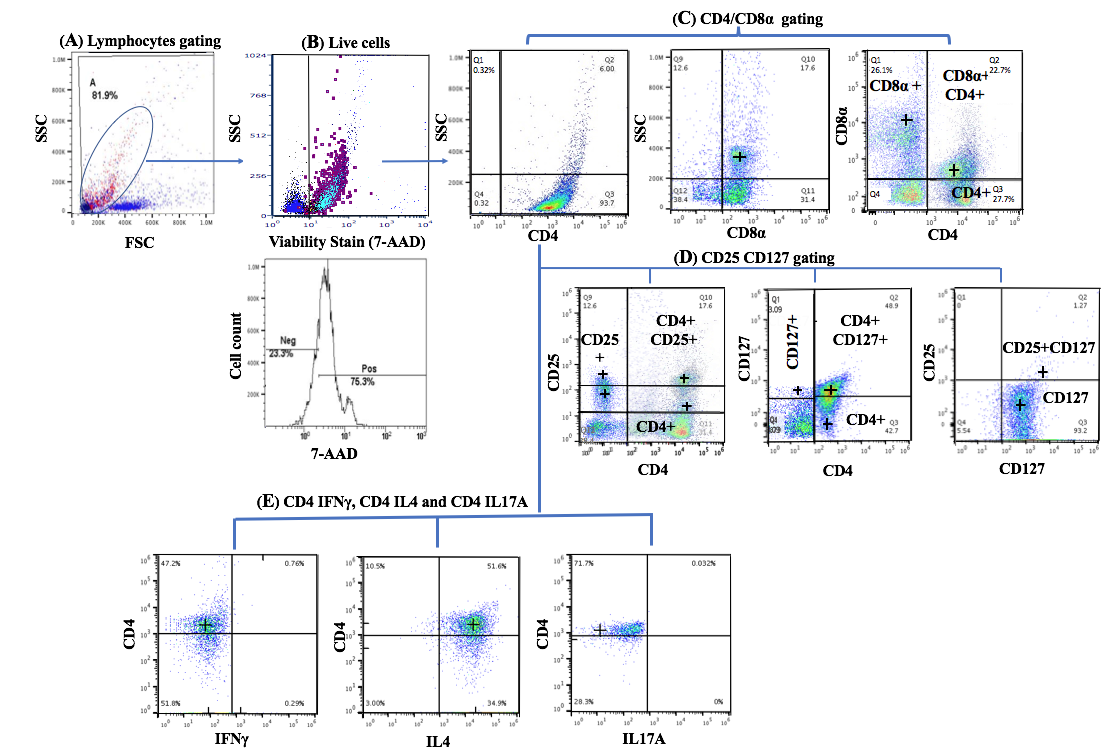
**

**Supplemental Figure S3**

**
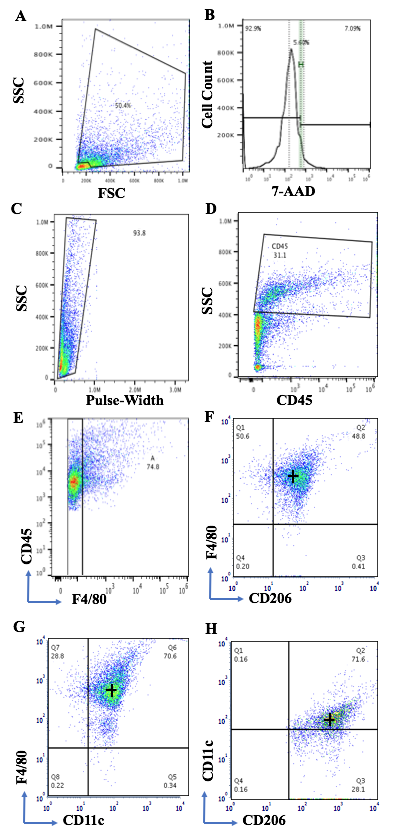
**

**Supplemental Figure S4**

**
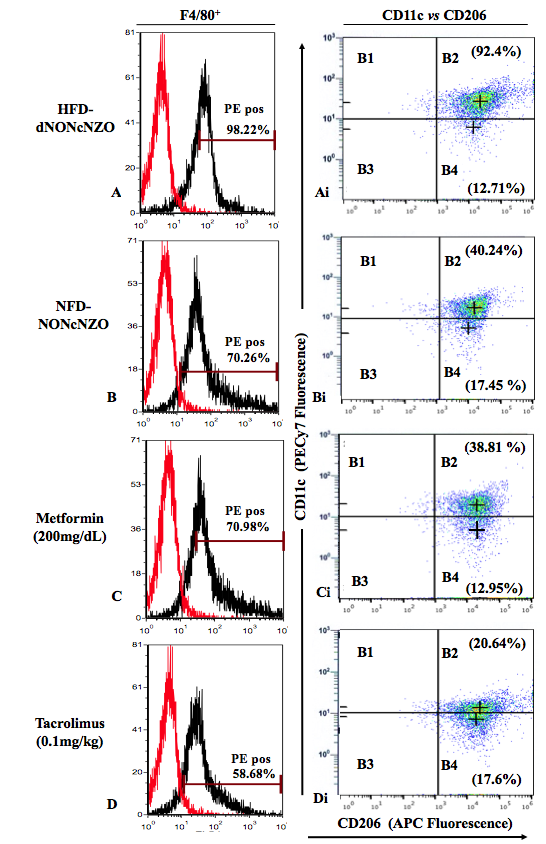
**

**Supplemental Figure S5**

**
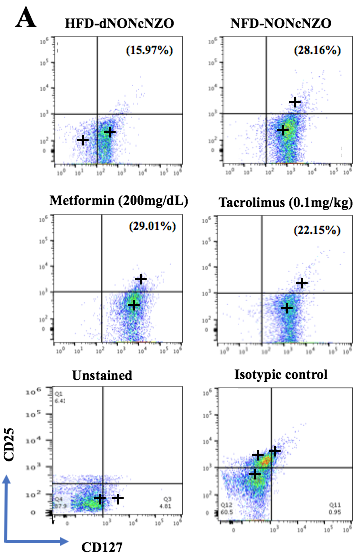
**

**Supplemental Figure S5 (continued)**

**
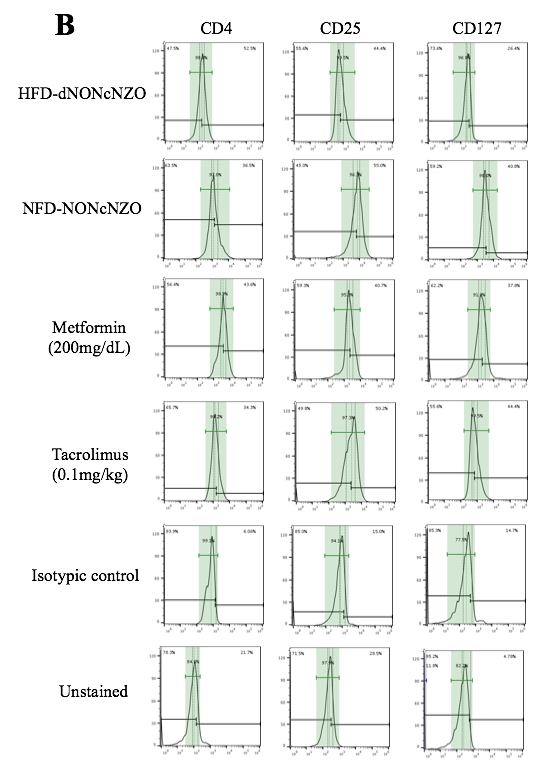
**

**Supplemental Figure S6**

**
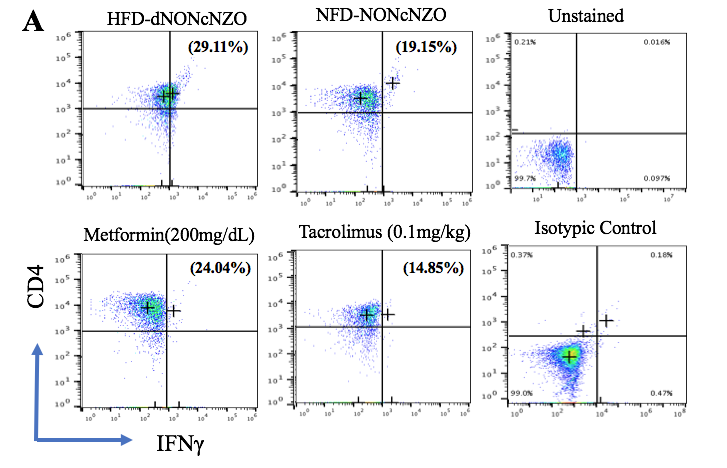
**

**
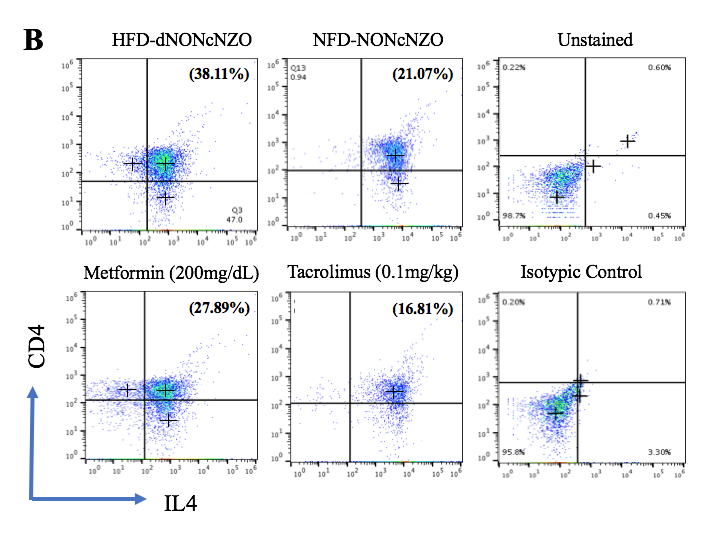
**

**Supplemental Figure S6 (continued)**

**
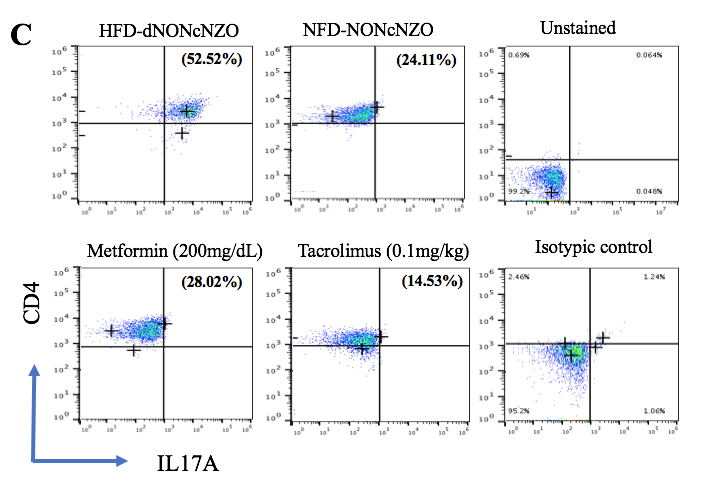
**

**Supplemental Figure S6 (continued)**


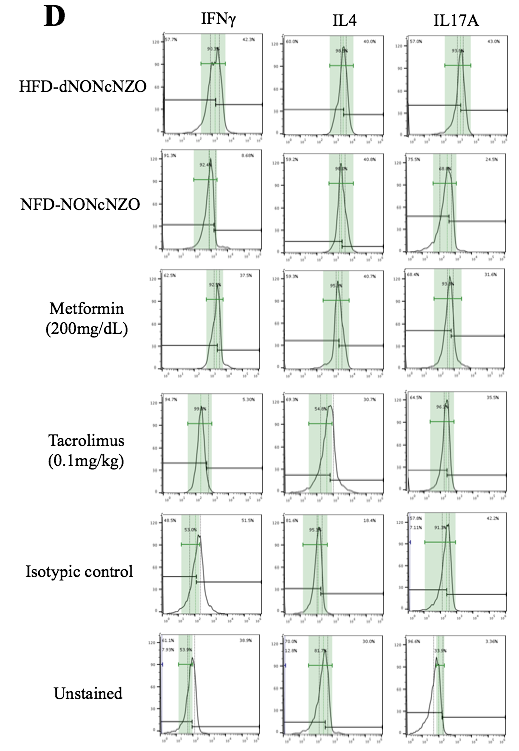


**Supplemental Figure S7**

**
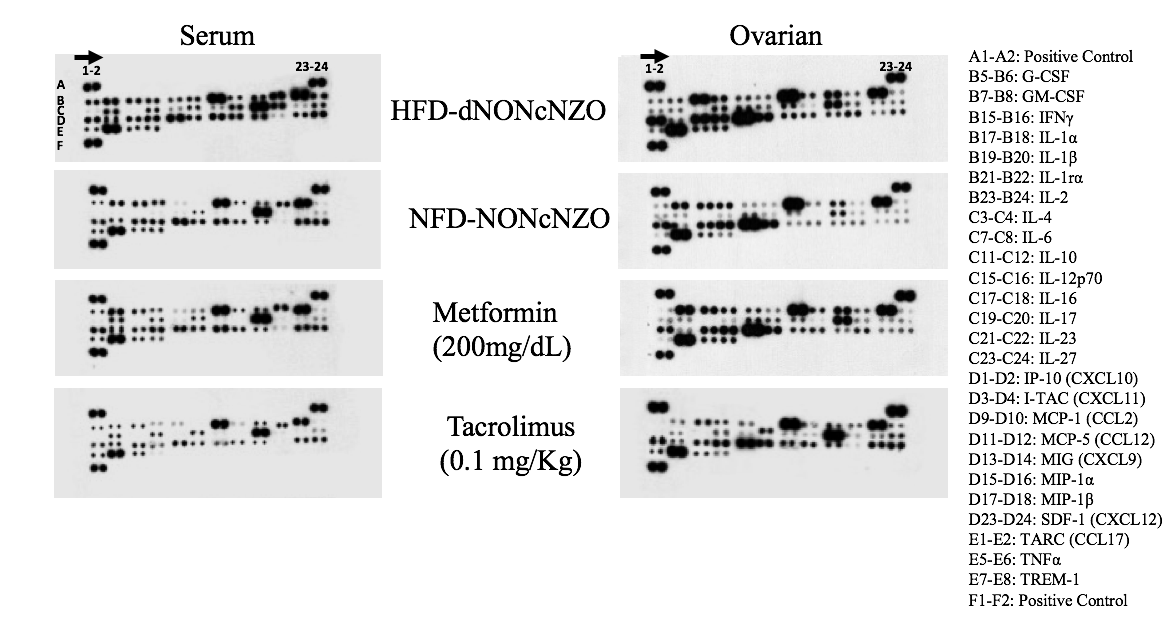
**
